# Proteomic insights into extinction memory deficits in stress-susceptible female rats

**DOI:** 10.3389/fnbeh.2025.1703714

**Published:** 2026-01-13

**Authors:** Nashaly Irizarry-Méndez, Yelitza Acosta-Pierantoni, Alondra Diaz-Vazquez, Anixa Hernández, Maria Colón, Eduardo L. Tosado-Rodríguez, Yadira M. Cantres-Rosario, Abiel Roche-Lima, Ana E. Rodríguez-De Jesús, Loyda M. Meléndez, James T. Porter

**Affiliations:** 1 Department of Basic Sciences, Ponce Research Institute, Ponce Health Sciences University, Ponce, Puerto Rico; 2 Integrated Informatics Services Research Capacity Core, Center for Collaborative Research in Health Disparities (CCRHD), University of Puerto Rico Medical Sciences, San Juan, Puerto Rico; 3 School of Dental Medicine, Universidad Ana G. Méndez, Gurabo, PR, United States; 4 Translational Proteomics Center, Research Capacity Core, Center for Collaborative Research in Health Disparities, Academic Affairs Deanship, University of Puerto Rico Medical Sciences, San Juan, Puerto Rico; 5 Department of Microbiology and Medical Zoology, University of Puerto Rico Medical Sciences, San Juan, PR, United States

**Keywords:** fear extinction, infralimbic cortex, neurogranin, quantitative proteomics, single prolonged stress, stress susceptibility, tau protein

## Abstract

Stress exposure can disrupt fear extinction, which is a hallmark of some stress-related disorders. The underlying molecular mechanisms of impaired extinction, especially in females, remain poorly understood. In this study, we investigated proteomics changes in the infralimbic cortex, a region critical for fear suppression, in female rats exposed to single prolonged stress (SPS). One week after SPS exposure, adult female rats underwent auditory fear conditioning and extinction training and were classified as susceptible or resilient based on their extinction performance. Quantitative proteomics using tandem mass tag labeling combined with bioinformatics analysis identified distinct proteins and pathways differentiating the groups. Susceptible rats displayed unique proteomic profiles in the infralimbic cortex. Several of the 53 differentially expressed proteins are associated with synaptic plasticity and memory, including neurogranin and microtubule-associated protein tau (MAPT). Pathway enrichment analysis identified alterations in synaptogenesis, clathrin-mediated endocytosis, calcium signaling, and chaperone-mediated autophagy. Functional validation using AAV-shRNA knockdown of neurogranin or MAPT in CAMKIIα-expressing neurons of the infralimbic cortex improved extinction memory in SPS-exposed animals. Our findings suggest that dysregulated protein expression in the infralimbic cortex contributes to impaired extinction memory and traumatic stress susceptibility in female rats, offering insight into the neurobiological mechanisms underlying vulnerability to stress-related disorders.

## 1 Introduction

Learned fear serves as an adaptive mechanism for survival, enabling individuals to recognize and respond to threats. However, when individuals cannot regulate fear responses, trauma-related disorders can develop. Exposure to stress can significantly impair recovery from trauma by disrupting fear extinction, a learning process that plays an essential role in suppressing fear memories (Lancaster et al., 2016; Maren and Holmes, 2016). Although women are nearly twice as likely as men to develop trauma-related disorders after experiencing trauma (National Center for PTSD, 2018), individuals exposed to the same traumatic experiences do not respond equally. While some develop adverse physiological and behavioral effects, others remain resilient (Benjet et al., 2016; Koenen et al., 2017). The neurobiological mechanisms underlying stress susceptibility in females remain poorly understood (Hu et al., 2017; Olff, 2017; National Center for PTSD, 2018). This gap is primarily due to the lack of clinical and preclinical studies that include female subjects (Beery and Zucker, 2011). Understanding the factors that contribute to differences in stress susceptibility in females is essential, given that females are disproportionately affected by stress-related disorders and remain understudied in neuroscience research.

Literature review indicates that molecular changes in the fear circuitry contribute to determining susceptibility to trauma in male rodents (Al Jowf et al., 2021). One important brain structure involved in the development of trauma-related disorders is the ventromedial prefrontal cortex, which corresponds to the infralimbic cortex (IL) in rodents. The IL plays a critical role in suppressing conditioned fear responses and facilitating extinction memory (Likhtik, 2005; Corcoran and Quirk, 2007; Sierra-Mercado et al., 2011). Functional disruption of the IL produces fear extinction deficits (Etkin and Wager, 2007), and inactivation of IL before extinction training impairs the retrieval of extinction (Sierra-Mercado et al., 2006), implying that IL activation is essential for the plasticity underlying fear extinction memory.

To investigate the impact of traumatic stress on brain physiology and animal behavior, researchers have used the single prolonged stress (SPS) paradigm in rodents. SPS induces heightened fear conditioning, impaired fear extinction, decreased activity of the IL, and enhanced glucocorticoid levels (Liberzon et al., 1997; Knox et al., 2012; Piggott et al., 2019; Nawreen et al., 2021), mimicking features of some trauma-related disorders. However, most SPS studies have been conducted in male rodents, with only a few including females (Keller et al., 2015; Pooley et al., 2018a; Pooley et al., 2018b; Mancini et al., 2021; Collins et al., 2023; Torres-Rodríguez et al., 2023; Torres-Rodriguez et al., 2023), and none have examined the effects of SPS on the IL cortex in female rodents.

Given that IL dysfunction is associated with impaired fear extinction (Etkin and Wager, 2007) and its activity is altered by SPS exposure (Nawreen et al., 2021), examining protein changes in this region may reveal neurobiological mechanisms underlying individual variability in extinction retrieval after SPS exposure. Quantitative proteomics offers an unbiased approach to identify proteins and pathways associated with behavioral phenotypes (Cookson, 2019; Shansky and Murphy, 2021). By comparing the IL proteomic profiles of female rats classified as susceptible or resilient following SPS exposure, we aimed to uncover molecular markers of stress vulnerability that may inform future therapeutic strategies for extinction deficits.

In this study, we used tandem mass tag (TMT)-based quantitative proteomics to analyze protein expression in the IL of SPS-exposed female rats, which were classified as resilient or susceptible based on their extinction retention performance. Among the differentially expressed proteins, neurogranin and microtubule-associated protein tau (MAPT) were significantly upregulated in susceptible animals. Neurogranin regulates calmodulin availability and calcium signaling at the postsynaptic level, contributing to synaptic plasticity and learning (Zhong et al., 2009), while MAPT stabilizes microtubules and is implicated in memory dysfunction and neurodegeneration (Zhang et al., 2016). To test their functional relevance, we used AAV-shRNA to selectively reduce neurogranin or MAPT in CAMKIIα-expressing neurons in the IL. This manipulation significantly improved extinction memory in SPS-exposed female rats, supporting a causal role for these proteins in stress-induced extinction impairment. These findings aim to improve our understanding of trauma vulnerability in females and support the development of targeted interventions for trauma-related disorders.

## 2 Materials and methods

### 2.1 Animal subjects

Adult female Sprague–Dawley rats were obtained from the Ponce Health Sciences University colony. They were housed two per cage and maintained under standard conditions with a 12 h light/dark cycle with free access to food and water. The Ponce Health Sciences University Institutional Animal Care and Use Committee (IACUC) approved all the animal work (IACUC No. 2203000877 and IACUC No. 2411144063). All research followed the National Institutes of Health’s Guide for the Care and Use of Laboratory Animals.

### 2.2 Single prolonged stress

Sprague–Dawley female rats, approximately postnatal day 60, were subjected to the SPS protocol as described by Knox et al. (2012) and colleagues and Liberzon and Young (1997) and colleagues. Each animal underwent SPS individually. The SPS procedure began with a 2-h restraint stress using a disposable rodent restrainer (DecapiCone®, Cat. No. DC-200). This was immediately followed by a 20-min swim in a cylinder (20 cm diameter X 45 cm tall) containing tap water at 24 °C. After the swim, the rats were allowed a 10-min recovery period in a cage placed under a soft white 60-watt bulb to provide gentle heat. Subsequently, animals were placed in a chamber (16 cm x 16 cm) containing a disposable paper towel impregnated with ethyl ether (Millipore Corporation, Cat. No. EX0185-8) inside a chemical hood. To prevent direct contact with the paper towel, rats were placed on a mesh platform inside the chamber, ensuring exposure only to the vapors. Exposure continued until animals were unresponsive to external stimuli (approximately 4 min). After completing SPS, animals were pair-housed with free access to food and water and left undisturbed for 7 days before behavioral testing.

### 2.3 Auditory fear conditioning and extinction training

Seven days after SPS, animals were exposed to classic Pavlovian fear conditioning to test their ability to acquire and extinguish a fear memory associated with an auditory cue. All behavioral procedures were conducted in a clear 25 × 29 × 28 cm Plexiglas chamber (ID#46002, Ugo Basile, Gemonio, Italy). The floor in the chamber consisted of stainless-steel bars that provided the shocks (0.50 mA, 0.5-s duration). The chamber was housed in a noise-isolating box, which had a video camera for recording the behavior. Between trials, chambers were cleaned with a 70% ethanol solution. The fear response was measured as the time the rats spent immobile when a tone (30 s) was played. All videos were recorded and analyzed using the Any-Maze Software. The behavioral procedure consisted of fear conditioning (Day 8), extinction (Day 9), and extinction recall (Day 10). On the first day, the rats received one unpaired tone (30 s, 1 kHz, 80 dB) followed by five tone-shock pairings in context A. Each tone-shock pairing was separated by a 3-min intertrial interval (ITI). During extinction training, rats were exposed to 14 tones (30 s, 1 kHz, 80 dB) separated by a 3-min ITI in context B. The following day, rats received two tones (30 s, 1 kHz, 80 dB) during the extinction recall test in context B. In context B, the visual and tactile cues were changed. Lack of movement or freezing was quantified as the fear response. All behavioral recordings were reviewed twice for accuracy. Periods in which animals were sleeping were assigned a freezing score of 0%, as sleep was not considered a fear-related response. Animals were euthanized 90 min after the extinction recall test, with an overdose of intraperitoneal 65 mg/kg Euthanasia-III Solution (Pentobarbital Sodium, Phenytoin Sodium, MED-PHARMEX™). After presenting a lack of response to touch, vaginal smears were collected, and animals were transcardially perfused using 0.9% saline. For proteomics analyses, brains were rapidly collected and stored at −80 °C until further processing. Animals used for histological analyses were perfused with saline 0.9%, followed by 10% Buffered Formalin (VWR, 89370-094). Brains were extracted and immersed in Falcon tubes containing approximately 7 mL of 30% sucrose–formalin solution. Samples were maintained at 4 °C until the tissue sank, indicating complete sucrose saturation.

### 2.4 Extinction retention index (ERI)

The ERI was calculated to assign individual animals to the resilient or susceptible group based on their ability to learn and retrieve extinction. Animals within the top 33% ERI score were classified as resilient, and the animals within the bottom 33% ERI score were classified as susceptible (Pan et al., 2022). The ERI was calculated with the following formula: [(Avg Ext 1,2) - (Avg Ext Recall 1,2)]/(Max Fear acquired COND) × 100. The ERI calculation has been used in 37 studies, including humans and rodents (Lonsdorf et al., 2019). This index expresses extinction retention responses as a percentage of acquisition responses. The extinction learning index (ELI) was also compared between groups. ELI was calculated with the following formula: [(Avg Ext 1,2) - (Avg Ext 13,14)]/(Max Fear acquired COND) × 100 (Pan et al., 2022).

### 2.5 Preparation of protein and protein determination

At the time of euthanasia, the brain was collected, and tissue punches (8 mg to 12 mg) from the IL were extracted. To enrich membrane proteins, we followed the method described by Neuner et al. (2015) and colleagues. First, we homogenized the samples in a lysis buffer (0.32 M sucrose, 3 mM HEPES, pH 7.4) containing commercial phosphatase inhibitors (Phosphatase Inhibitor Set I, 1:100, Cat. No. 524624; Phosphatase Inhibitor Set II, 1:100, Cat. No. 524625) and a protease inhibitor (Protease Inhibitor Cocktail Set III, EDTA-Free, 1:200, Calbiochem, Cat. No. 539134). This mixture was combined with a high-salt buffer (2 M NaCl, 10 mM HEPES, 1 mM EDTA, pH 7.4) and centrifuged at 16,000 g for 20 min at 4 °C to obtain the whole protein fraction. The remaining pellet was then homogenized in carbonate buffer (0.1 M Na₂CO₃, 1 mM EDTA, pH 11.3) and incubated for 30 min at 4 °C to separate membrane fractions from free cytosolic proteins and organelles. After incubation, we centrifuged the samples again at 16,000 g for 20 min at 4 °C. Next, to extract membrane proteins from the lipid bilayer, we re-dissolved the pellet in ice-cold urea buffer (5 M urea, 100 mM NaCl, 10 mM HEPES, 1 mM EDTA, pH 7.4) and centrifuged at 16,000 g for 20 min at 4 °C to isolate the postsynaptic density. The remaining pellet was disrupted and washed twice with ice-cold Tris/HCl buffer (pH 7.6) and centrifuged each time at 16,000 g for 20 min at 4 °C. Finally, we dissolved the pellet in 2% SDT-lysis buffer (4% SDS, 100 mM dithiothreitol in 0.1 M Tris/HCl buffer, pH 7.6) containing Phosphatase Inhibitor Sets I/II and Protease Inhibitor Cocktail III. Whole protein, cytosolic, and membrane fractions were combined and used for subsequent analysis. A detergent-compatible Bicinchoninic acid assay (Bio-Rad, Hercules, CA, USA, Cat. No. 500116) was performed for total protein quantification according to the manufacturer’s instructions. Samples were assayed in technical replicates and read at 570 nm in a Varioskan Flash Spectral Reader (Thermo Fisher Scientific, Mount Prospect, IL, USA). Protein samples were aliquoted and stored at −20 °C until TMT analyses.

### 2.6 Preparation of protein samples for TMT labeling

A total of 10 samples were randomly selected for quantitative proteomics analysis (5 resilient rats and 5 susceptible rats). Briefly, 100 μg of proteins were concentrated by incubating with 10% SDS and acetone overnight at −20 °C; the next day, after centrifugation at 10,000 g, pellets were resuspended in sample buffer (95% Laemli/ 5% B-mercaptoethanol) at a concentration of 2 μg/μL. Subsequently, samples were heated at 70 °C and loaded into precast TGX mini-protean gels for SDS-PAGE (Bio-Rad, 12% Mini-PROTEAN® TGX™ Precast Protein Gels, 10-well, 50 μL Cat. #4561044). The gels ran for 15 min at 150 V, fixed and Coomassie stained. Gel pieces were cut and de-stained by incubation using a solution of 50% acetonitrile and 50 mM ammonium bicarbonate at 37 °C for 2 to 3 h. Thereafter, they were reduced with 25 mM dithiothreitol in 50 mM ammonium bicarbonate for 30 min at 55 °C and alkylated with 10 mM iodoacetamide at room temperature in the dark. Samples were then digested at 37 °C overnight with a grade-modified trypsin solution in 50 mM ammonium bicarbonate (Promega, Madison, WI, USA). The Trypsin/Protein ratio used for optimal digestion was 1:50. The next day, digested peptides were extracted from the gel pieces using a mixture of 50% acetonitrile and 2.5% formic acid in water. Extracted peptides were dried and stored at −20 °C for TMT labeling (Supplementary Table 1).

### 2.7 TMT Labeling

Dried extracted samples were reconstituted in 100 mM TEAB (triethyl ammonium bicarbonate) and labeled with the TMT 11plex labeling reagents. Following one-hour incubation with occasional vortexing and a quenching step of 15 min, equal amounts of each sample per kit were mixed to generate a final pool that was later submitted to fractionation. This method was performed for each final pool per TMT kit using the Pierce High pH Reversed-Phase Peptide Fractionation Kit (REF 89875) and following the manufacturer’s instructions. Briefly, the column was conditioned twice using acetonitrile, centrifuged at 5,000 × g for 2 min, and the steps were repeated using 0.1% Trifluoroacetic acid (TFA). Each TMT-labeled pool was reconstituted in 300 μL of 0.1% TFA, loaded onto the column, and washed to remove salt contaminants or any unbound TMT reagent. The clean pooled sample was then eluted into 8 different vials using a series of elution solutions with different Acetonitrile/0.1% Triethylamine percentages. Elution solutions are specified in the manufacturer’s protocol. Fractions were dried and reconstituted for mass spectrometry analysis using 0.1% formic acid in water (Buffer A). A small portion (3 μL) was transferred to a special sample vial for injection into the instrument.

### 2.8 Liquid chromatography/mass spectrometry (LC–MS/MS) protein identification and quantitative analysis

The liquid chromatography and mass spectrometry analysis was performed using an Easy nLC 1,200 (Thermo Fisher Scientific) coupled to the Q-Exactive Plus Orbitrap (Thermo Fisher Scientific). A PicoChip chromatographic column (New Objective) was used with the following specifications: H354 REPROSIL-Pur C18-AQ 3–5 μm, 120–300 Å, and 105 mm bed length. Mobile phases for the gradient consisted of 0.1% formic acid in water (Buffer A) and 0.1% formic acid in 80% acetonitrile (Buffer B). Peptide separation was obtained using a gradient of 7–25% of Buffer B for 102 min, 25–60% of Buffer B for 20 min, and 60–95% Buffer B for 6 min. Making a total gradient time of 128 min at a flow rate of 300 nL/min, a maximum pressure of 300 bars, and an injection volume of 2 μL per sample. The Q-Exactive Plus operates in positive polarity mode and data-dependent mode. The full scan (MS1) was measured over the range of 375 to 1,400 at a resolution of 70,000. The MS2 analysis was configured to select the ten (10) most intense ions (Top10) for HCD fragmentation with a resolution of 35,000, an AGC target of 1e5, and a 1.2 m/z isolation window. Collision energy was set to 32. A dynamic exclusion parameter was set for 30 s.

### 2.9 Database search

Mass spectrometric raw data files were analyzed using Proteome Discoverer (PD) software version 2.5. Files were searched against a *Rattus norvegicus* database obtained from the PD protein knowledge base (tax ID = 10,116). Sequest HT was used for the search. Trypsin was specified as the enzyme for proteolysis with a maximum of 2 mixed cleavage sites. Precursor mass tolerance was set to 20 ppm, and Fragment mass tolerance was set to 0.5 Da. The modifications included were a dynamic modification for oxidation +15.995 Da (M), a static modification of +57.021 Da (C), and static modifications from the TMT reagents +229.163 Da (Any N Term, K). Channel 126 was marked as the control channel. The TMT certificate of analysis (Lot: XL338485) was used to correct for isotopic impurities of reporter ions. The percolator node was included for Target/Decoy selection and FDR targets were set to 0.01 (Strict) and 0.05 (Relaxed). These results were submitted to further bioinformatic analyses detailed below.

### 2.10 Differentially abundant protein identification and ingenuity pathway analysis

Protein expression analysis was conducted on eight biological samples, comprising four control samples classified as resilient (good extinction retention index) and four case samples classified as susceptible (poor extinction retention index). A total of 1,624 proteins were identified and subjected to statistical differential abundance analysis using the MetaboAnalyst 6.0 online platform (Costanzo et al., 2022; Ewald et al., 2024; Pang et al., 2024). Proteins were considered significantly dysregulated if they exhibited a fold change ≥ |1.5| and a *p*-value ≤ 0.05. Comparative analyses between susceptible and resilient groups were performed to identify differentially abundant proteins. Sample composition was assessed using PCA, while differentially abundant proteins were visualized using volcano plots, both generated via MetaboAnalyst 6.0. Protein annotations, including names and functional descriptions, were retrieved using UniProt accession identifiers. Pathway enrichment analysis was performed using Ingenuity Pathway Analysis (IPA, version 22.0.2, QIAGEN Digital Insights) to explore the functional implications of differentially expressed proteins. Canonical pathways, diseases, and functional associations were identified through IPA’s Ingenuity Core Analysis. Pathways were considered significantly enriched at –log10(*p*-value) ≥ 1.30 (*p* ≤ 0.05), while a threshold of *p* ≤ 0.05 was applied for disease and function analyses. A horizontal bar plot was generated to visualize the Z-score distribution of canonical pathways, illustrating their relative activation or inhibition. Z-scores were derived from pathway analysis and normalized to reflect the intensity of activation, represented through color gradients. The plot was constructed using Matplotlib (Hunter, 2007) and Seaborn (Waskom, 2021). Key canonical pathways of interest included Synaptogenesis, Clathrin-Mediated Endocytosis, Chaperone-Mediated Autophagy, Calcium Signaling, and 14-3-3-Mediated Signaling. Additionally, interaction network analysis was conducted for 10 selected proteins of interest (CD81, CD9, GAP43, MAP2, MAP6, MAPT, MYADM, SYNPO, CALM3, NGRN) using IPA, with our dataset serving as the reference. Detailed pathway diagrams were generated to visualize molecular interactions and regulatory networks associated with these proteins.

### 2.11 Immunohistochemistry

Immunohistochemical staining was performed on free-floating coronal brain sections (30 μm) prepared from fixed tissue with a cryostat. Sections were collected into multi-well plates containing 1X PBS and divided into rostral (Bregma 4.20 mm), medial (Bregma 3.00 mm), and caudal (Bregma 2.76 mm) IL regions. On Day 1, sections were transferred to Tris-Saline buffer (TS) (84.4 mM Trizma HCl, 16.51 mM Trizma base, 154.00 mM NaCl) and washed three times for 10 min on a shaker. Antigen retrieval was performed by incubating sections in citrate-EDTA buffer (10 mM citric acid, 2 mM EDTA, 0.005% Tween-20, pH 6.2) at 80 °C for 30 min, followed by cooling at room temperature for 20 min. Sections were then washed twice with TS (2 min each) and treated with 3% H₂O₂ for 30 min to quench endogenous peroxidase activity, followed by two washes with TS (10 min each). Blocking was carried out for 1 hour in 5% bovine serum albumin (BSA Fraction V, pH 5.2, VWR,0175) with 0.05% Triton X-100 (Sigma T-8787) in TS. Sections were then incubated overnight at 4 °C with primary antibodies diluted in 1% BSA and 0.05% Triton X-100 in TS: anti-Neurogranin (Abcam, ab217672; 1:20,000), anti-Tau (Santa Cruz, sc32274; 1:3,500). On Day 2, sections were washed three times (10 min each) in TS, then incubated for 1 hour at room temperature with biotinylated secondary antibodies (Vector Labs; BA-1000 for anti-rabbit and BA-9200 for anti-mouse) diluted in 1% BSA. An avidin-biotin complex (ABC) solution (Elite Vectastain PK-6100) was prepared at half the manufacturer’s recommended concentration and added for 45 min after three washes (5 min each). During ABC incubation, the DAB substrate was prepared by dissolving two DAB tablets (MP Biomedicals, MP 980681; 5 mg each) in 45 mL of TS with 160 μL of 100 mM NiCl₂. Activation was achieved by adding 4.5 μL of 30% H₂O₂ immediately before use. Sections were exposed to DAB for a predetermined time (neurogranin 3 min; tau 6 min), washed twice (10 min each) in TS, and mounted on charged slides to air-dry overnight. The next day, slides were dehydrated through graded alcohol (70, 80, 95, and 100%; 1 min each), cleared in xylene substitute (1 min), and cover slipped with Cytoseal. DAB-stained immunohistochemistry images were semi-quantitatively analyzed using ImageJ (National Institutes of Health).^1^ Images were converted to 8-bit grayscale and inverted so that areas of stronger DAB staining appeared brighter. Original color images were kept separately for anatomical reference. Measurement parameters were set to include area and integrated density. Consistent thresholding was applied to isolate the DAB-positive signal, using control sections (secondary without primary antibody) to exclude background staining. Threshold values were recorded and uniformly applied across all slices to ensure reproducibility. Finally, we calculated the average values and standard error of the mean for the integrated density to compare staining levels between groups.

### 2.12 Short hairpin RNA constructs

For protein knockdown experiments, we used adeno-associated viral vectors (AAV9) encoding short hairpin RNA (shRNA) constructs under the control of a CAMKIIα promoter with mCherry as a fluorescent reporter gene to selectively reduce either neurogranin or MAPT in CAMKIIα-expressing neurons. To target the rat neurogranin, the vector contained three different shRNA sequences targeting the neurogranin mRNA (Clone 1: ACATGGCGAGGAAGAAGATAAA, Clone 2: CTCCCGCTCTCCTTTGTTTATG, Clone 3: CTCCAAGCCAGACGACGATATT).^2^ The shRNA targeting the rat MAPT was driven by a CAMKIIα promoter with mCherry as a fluorescent reporter gene. The AAV9 construct contained three different shRNA sequences targeting the MAPT mRNA (Clone 1: CTGTAAGGATGAGGCCTTTAAA, Clone 2: GCGGCAAGGTGCAGATAATTAA, Clone 3: AGACAGACCATGGAGCAGAAAT).^3^ As a negative control, we used a scramble shRNA construct with the following sequences (Clone 1: ACCTAAGGTTAAGTCGCCCTCG, Clone 2: CCAACAAGATGAAGAGCACCAA, Clone 3: GGCCGCGATTAGGCTGTTATAA), also driven by the CAMKIIα promoter with mCherry as a fluorescent reporter gene.^4^ As a second control vector, we used an AAV5 vector expressing channelrhodopsin driven by CAMKII promoter with EYFP as a fluorescent reporter.^5^ The AAV constructs had a viral titer of more than 10^13^ GC per milliliter (GC/mL) as determined by quantitative PCR performed by the vendors.

### 2.13 Stereotaxic surgery

At the time of surgery, adult female rats (P60) were placed on a stereotaxic apparatus, injected with ketolorac (5 mg/kg, i.p.), and anesthetized with isoflurane (3–4%). A volume of 0.1 μL per hemisphere of an AAV vector expressing either neurogranin-shRNA, MAPT-shRNA, a Scramble RNA, or channelrhodopsin, was injected bilaterally into the IL (3.0 mm AP, ± 0.05 mm ML from bregma, − 4.8 mm DV from dura) using a Hamilton Neurosyringe at a rate of 0.05 uL/min. The syringe was lowered to −4.8 mm DV and the retracted to −4.7 mm to create a small pocket for injection. This 0.1 mm retraction step minimizes backflow and ensures consistent deposition of the viral particles at the target site. Following infusion, the syringe remained in place for an additional 10 min to allow viral diffusion further reduce backflow. Then, we removed the syringe and sealed the incision site with stitches. After a 30-day recovery period, to ensure adequate viral expression, rats were exposed to the following behavioral tests: SPS, approach task in an open field arena, auditory fear conditioning, extinction training, and extinction recall. Due to reduced extinction learning observed after surgery in our previous work (Soler-Cedeño et al., 2019; Torres-Rodríguez et al., 2023), rats that underwent surgery received shocks of 0.44 mA (0.5-s duration) and two extinction sessions separated by 4 h to ensure adequate extinction acquisition prior to recall testing. All behavioral recordings were reviewed twice for accuracy. Periods in which animals were sleeping were assigned a freezing score of 0%, as sleep was not considered a fear-related response. Animals were euthanized 90 min after the extinction recall test, with an intraperitoneal overdose of 65 mg/kg Euthanasia-III Solution (Pentobarbital Sodium, Phenytoin Sodium, MED-PHARMEX™). After presenting a lack of stimuli to touch, vaginal smears were collected, and animals were transcardially perfused using 0.9% saline, followed by 10% Buffered Formalin (VWR, 89370–094). The brain was extracted and placed in a Falcon tube with approximately 7 mL of 30% Sucrose Formalin solution and left in the refrigerator until it sank to ensure sucrose saturation. Brains were removed from the solution, and coronal sections of 30 μm were done using a Leica CM1520 cryostat to confirm viral efficiency and correct infusion site. Animals with viral expression in the prelimbic or dorsal peduncular cortex rather than the IL were excluded from analysis. Animals showing unilateral expression in the IL were included in the analysis.

### 2.14 Approach test in the open field arena

Each rat was individually placed in an open field arena (100 cm × 100 cm floor, 40 cm high walls) constructed from wood and coated with black acrylic. A transparent sphere was positioned at the center of the arena to serve as the novel object. The test was conducted under red light conditions, and behavior was video-tracked for 5 min using ANY-maze Software 7.4 (Stoelting Co., Wood Dale, IL, USA). Between trials, the arena and object were cleaned with 70% ethanol to remove olfactory cues. The following parameters were measured: total distance traveled, time spent in the periphery (thigmotaxis), time spent in the center zone, and time spent interacting with the object. The center zone was defined as the central (30 cm × 30 cm) area of the arena, while the periphery comprised the remaining area near the walls. Zone classification in ANY-Maze was based on the position of the rat’s body center, whereas object interaction was defined by the proximity of the head to the object.

### 2.15 Immunofluorescence staining

Immunofluorescence staining was performed on free-floating coronal brain sections (30 μm) previously prepared from fixed tissue using a Leica CM1520 cryostat. All steps were carried out at room temperature on a rocking platform unless otherwise noted. Sections were first washed in phosphate-buffered saline (PBS) for three 10-min intervals to remove excess fixative. Non-specific binding was blocked by incubating the sections for 2 h in a blocking solution containing 10% normal goat serum (NGS) and 0.3% Triton X-100 in PBS. Following blocking, sections were incubated for 24 h at 4 °C with primary antibodies diluted in PBS containing 1% NGS and 0.3% Triton X-100 against neurogranin (1:2,000; Cat No. ab217672, Abcam), Tau (1: 1,000; Cat No.sc32274, Santa Cruz), and CAMKII alpha (1:500, Cat No. MA1-048, Invitrogen). The next day, sections were washed again in PBS (3 × 10 min) before incubation with fluorescently labeled secondary antibodies in PBS with 1% NGS and 0.3% Triton X-100 for 1 h. For the secondary antibodies, we used Alexa Fluor® 488 goat anti-rabbit (A-21206, 1:200) (neurogranin and Tau) or 1:500 (CAMKII) and Alexa Fluor® 488 goat anti-mouse (A-11029, 1:200) from Life Technologies. After another set of PBS washes, nuclei were counterstained with DAPI (2 drops per 1 mL PBS) for 5 min. To prevent fluorescence photobleaching, Prolong Gold antifade reagent (Invitrogen P36934) was added before mounting slices. Images were captured using a Nikon Confocal Microscope A1 (Version 4.10) at 60X magnification.

*Z-stack analysis for neurogranin and Tau knockdown*: Maximum intensity projections of high-powered images (60x objective) were generated and merged into RGB composites (DAPI = blue, target protein = green, viral expression = red) using Fiji/ImageJ (NIH). Images were converted to 24-bit RGB format before analysis. An image was taken from three animals from each group, and two cells were pseudorandomly selected from each image by identifying an mCherry+ cell that also showed a clear DAPI-labeled nucleus and detectable green signal (immunolabeling of neurogranin or MAPT). Once a qualifying neuron was identified, a straight horizontal line (17 μm ) ROI was drawn across that single cell to extract fluorescence intensity. For each ROI, intensity profiles were obtained for the red, green, and blue channels using a custom ImageJ macro. Raw values allowed direct comparison of absolute fluorescence. Results were exported as CSV tables for further processing in Excel/GraphPad Prism. Data were presented as normalized green intensity values, calculated by dividing the raw green fluorescence intensity of each cell by the mean green intensity of infected cells in the control group.

*Cell quantification analysis*: For each animal, three non-overlapping fields of view were acquired from the IL area, avoiding tissue borders. To ensure unbiased quantification, only mCherry-positive cells that also exhibited clear nuclear staining (DAPI^+^) were included, excluding processes or debris. CAMKII-positive staining was quantified within the same focal plane using the DAPI-based inclusion criterion. Double-positive cells (mCherry^+^/CAMKII^+^) were identified in each field. Counts from the three images per animal were summed to generate a single value per animal. The percentage of infected neurons that were CAMKIIα-expressing neurons was calculated as (Double positive cells/total mCherry+ cells) × 100, expressed as %CAMKII^+^ among mCherry^+^ cells. Percentages calculated per animal were plotted as individual biological replicates using GraphPad Prism.

### 2.16 Statistical analysis

Freezing behavior (lack of movement) during each 30-s tone was measured using ANY-maze software. Data was analyzed with GraphPad Prism (Dotmatics). All datasets were first tested for normality. For Day 1 and Day 2 of auditory fear conditioning, a two-way repeated measures ANOVA was used. For comparisons between two groups, unpaired t-tests were used for normally distributed data, while Mann–Whitney U tests were applied to nonparametric data. For surgery experiments, a two-way repeated measures ANOVA was used for Day 1 and Day 2 of auditory fear conditioning and extinction. On Day 3 (Recall) and in the Open Field Test, one-way ANOVA followed by Tukey’s post hoc test was performed for parametric data. When data did not meet parametric assumptions, a Kruskal–Wallis test followed by Dunn’s post hoc test (uncorrected) was applied. Statistical significance was defined as *p* < 0.05. Data are presented as mean ± SEM (Standard Error of the Mean).

## 3 Results

### 3.1 Individual variability in fear extinction retention following single prolonged stress in female rats

**Figure 1:**
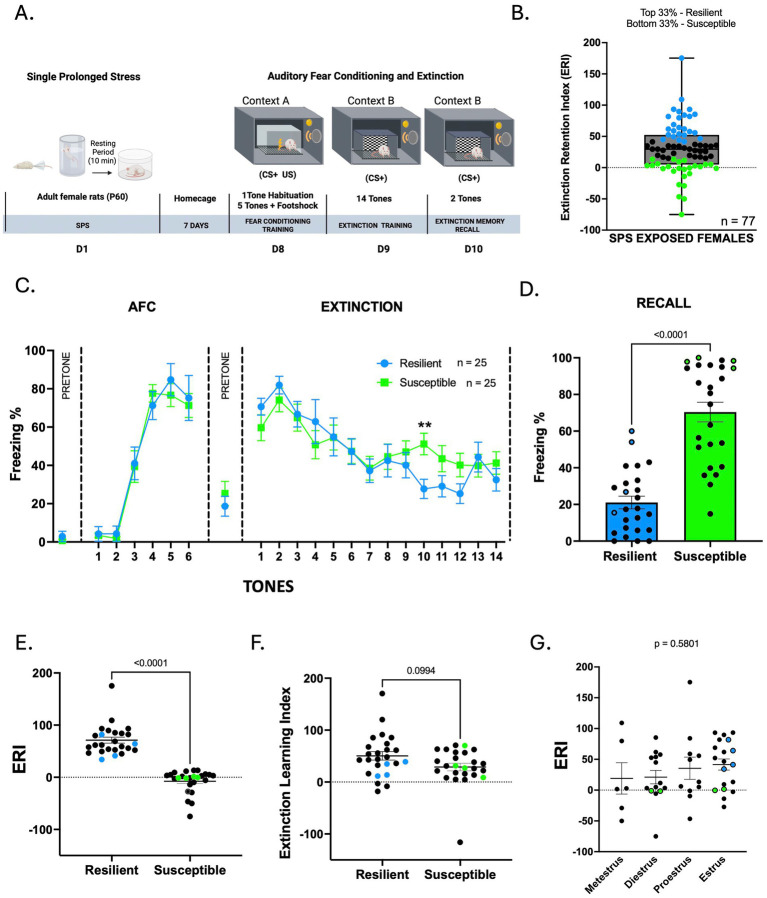
SPS exposure induced phenotypes associated with stress susceptibility and stress resilience. **(A)** Timeline for behavioral training and molecular assays in adult female rats. **(B)** ERI distribution of animals in this cohort was used to classify stress susceptible and resilient animals. **(C)** Freezing levels across auditory fear conditioning and extinction training in resilient (*n* = 25) and susceptible (*n* = 25) females exposed to SPS. Two-way ANOVA, uncorrected Fisher’s LSD *p* < 0.05. **(D)** Freezing levels during extinction recall in resilient and susceptible animals. Unpaired *T*-test, *p* < 0.05. **(E,F)** ERI and ELI of resilient and susceptible animals. Mann–Whitney test *p* < 0.05. Colored dots represent the animals selected for proteomic analysis. **(G)** Comparison of ERI with the estrous cycle during extinction recall. Data points represent 50 individual animals. One-way ANOVA, *p* < 0.05. Colored dots represent the animals selected for proteomic analysis. **(A)** was created with BioRender.com.

In the first set of experiments, we exposed adult female Sprague Dawley rats to SPS, followed by auditory fear conditioning and extinction (Figure 1). The extinction retention index (ERI) was used to quantify the persistence of conditioned fear responses. Animals were divided into two groups based on their ERI scores, with higher scores indicating resilience (reduced fear) and lower scores indicating susceptibility (persistent fear). Results showed the variability in the extent to which learned fear was extinguished across individuals (Figure 1B). These findings set the basis to investigate the molecular factors influencing these behavioral profiles.

Next, we examined the responses of the resilient and susceptible groups during auditory fear conditioning, extinction, and extinction recall (Figures 1C,D). A two-way repeated measures ANOVA showed a main effect of tone [*F*(6.466, 310.4) = 34.07, *p* < 0.001]. During fear conditioning, both groups exhibited similar levels of freezing to context A before the first tone (*p* = 0.4553) and to the first conditioning tone (*p* = 0.8490), as well as at the end of conditioning (*p* = 0.7699). The next day, when the animals were placed in context B, both groups displayed similar levels of contextual fear during the pretone period (*p* = 0.4205) and at the start of extinction training (EXT 1: *p* = 0.1842, EXT 2: *p* = 0.3166). The absence of significant differences in freezing behavior between the two groups during initial fear conditioning and extinction training indicates that both susceptible and resilient animals acquired and recalled fear memory similarly. By the end of the extinction training on day 2, both groups showed similar levels of freezing (*p* = 0.2983), suggesting that both groups learned extinction (Figure 1C). However, on day 3, susceptible animals showed significantly higher levels of freezing compared to the resilient ones (Mann–Whitney test, U = 48, *p* < 0.001), suggesting an impairment in extinction recall (Figure 1D).

Consistent with the separation of groups by the ERI, the ERIs of the susceptible females were lower than those of the resilient females (Mann–Whitney test, U = 0, *p* < 0.001) (Figure 1E). To determine whether poor extinction learning contributed to the impaired extinction recall in susceptible animals, we assessed the extinction learning index (ELI). Both groups exhibited similar ELI scores (Mann–Whitney test, U = 136, *p* = 0.2060), suggesting that they had comparable acquisition of extinction (Figure 1F). Next, we compared the ELI and ERI to evaluate differences in extinction acquisition and retention in resilient and susceptible animals. Additionally, we also compared recall performance with ERI to examine their relationship across stress subtypes. We analyzed these indices separately for resilient and susceptible animals. In resilient animals, ERI was greater than ELI (Wilcoxon test, W = 166.00, *p* = 0.0164), indicating better retention of extinction memory relative to extinction learning index (Supplementary Figures 1A,C). ERI was also significantly higher than recall freezing levels (Wilcoxon test, W = 305.00, *p* < 0.0001), suggesting a stronger retention index than freezing expression during recall. In contrast, susceptible animals showed the opposite pattern: ERI was significantly lower than ELI (Wilcoxon test, W = −272.00, *p* < 0.0014), and lower than recall freezing levels (Wilcoxon test, W = −325.00, *p* < 0.0001), suggesting deficits in extinction memory retention (Supplementary Figures 1B,D). Together, these results show that resilient animals maintain strong extinction memory, whereas susceptible animals exhibit marked retention deficits.

Finally, we assessed whether the estrous cycle on the day of extinction recall influenced susceptibility to develop impaired extinction. A one-way ANOVA showed no statistical difference between groups [*F*(3, 34) = 0.6612, *p* = 0.5801], suggesting that the estrous cycle stage on the day of extinction recall did not significantly impact the extinction retention index (Figure 1G).

### 3.2 Differentially expressed proteins in the IL of stress-susceptible and stress-resilient females

**Figure 2:**
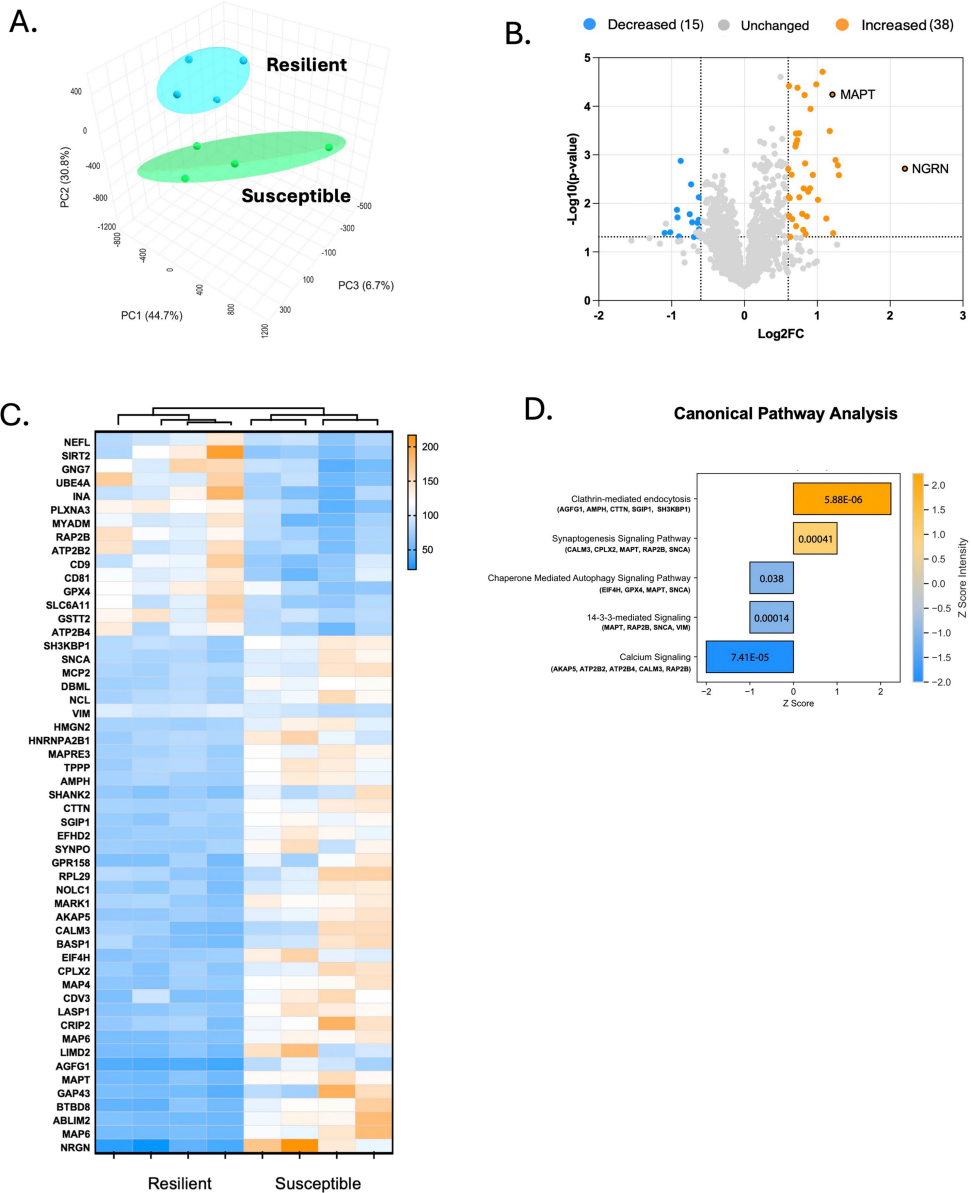
Differentially expressed proteins in stress susceptible and stress resilient rats. **(A)** PCA score plot showing differences between the stress susceptible group and the stress resilient group. PERMANOVA analysis *F*-value: 3.5602; *R*-squared: 0.3724; *p*-value (based on 999 permutations): *p* = 0.025. Each point represents individual animals of the susceptible (*n* = 4) and resilient (*n* = 4) groups. **(B)** Volcano plot of differentially expressed proteins in susceptible and resilient rats using the established criteria of a Fold Change ≥ |1.5| and *p*-value ≤ 0.05. Blue (decreased); Orange (increase); Unchanged (gray). **(C)** Hierarchical clustering analysis of differentially expressed proteins in the susceptible and resilient groups ordered by the magnitude of fold change. The *x*-axis represents each animal per group, and the *y*-axis represents the differentially expressed proteins. Higher relative abundance is indicated by orange and lower by blue. **(D)** Canonical pathways of differentially expressed proteins in the susceptible group versus the resilient group. Pathways are color-coded based on their *Z*-score, with negative *Z*-scores in blue and positive *Z*-scores in orange. The *p*-value for each pathway is indicated within the bars.

To identify proteins that might impact the differences in stress-susceptibility, we collected tissue punches from the IL of SPS-exposed females classified as resilient or susceptible. Using quantitative proteomics (TMT labeling), we identified a total of 1,624 proteins in the IL. Using principal component analysis (PCA) of the proteomic data, we determined whether stress subtypes (susceptible vs. resilient) corresponded to distinct protein profiles. We initially ran a PCA with five animals in each group and performed a permutational multivariate analysis of variance (PERMANOVA). This analysis showed an *F*-value of 2.2397, an R-squared of 0.21872, and a *p*-value of 0.122 (based on 999 permutations). One animal of each group fell outside the confidence ellipse in the PCA plot, identifying it as an outlier. We also examined the behavioral performance of these two animals. Although they were classified correctly based on their ERI, one animal showed unusually low freezing at the start of extinction suggesting impaired fear recall, while the other displayed weak fear conditioning during acquisition. These findings suggest that variability in behavioral phenotypes may influence the proteomic profiles observed in the IL. (Supplementary Figure 2). Therefore, we excluded these animals and conducted subsequent analyses with the remaining four animals per group. PCA showed two distinct clusters, indicating a clear separation of stress-susceptible and stress-resilient groups based on their proteomic profiles (Figure 2A). To validate the clustering observed in the PCA, we repeated the PERMANOVA analysis after removing the outliers. The analysis showed a significant effect of stress subtype on proteomic profiles (*F* = 3.5602, R^2^ = 0.3724, *p* = 0.025, based on 999 permutations), supporting the idea that stress-susceptible and stress-resilient animals exhibit distinct molecular profiles.

Applying the criterion of a fold change ≥ |1.5| and a *p*-value <0.05, we detected 53 differentially expressed proteins. Among these, 38 proteins were upregulated (Table 1), and 15 were downregulated in susceptible animals compared to resilient animals (Table 2). We further analyzed the significant proteins associated with stress responses and their subcellular localization. Most of the proteins were localized to the cytoplasm (24 out of 53), followed by the plasma membrane (18 out of 53) and the nucleus (10 out of 53). A single protein fell into the “other” category.

**Table 1:** Upregulated proteins showing increased abundance in susceptible animals compared to resilient animals.

Accession	Symbol	Entrez gene name	FC	*p*-value	Location
Q925Q9	SH3KBP1	SH3 domain-containing kinase-binding protein 1	1.5181	0.0019659	cytoplasm
P37377	SNCA	Alpha-synuclein	1.5271	0.0074254	cytoplasm
Q00566	MCP2	Methyl-CpG-binding protein 2	1.5278	0.017975	nucleus
Q9JHL4	DBNL	Drebrin-like protein	1.5286	3.81E-05	cytoplasm
P13383	NCL	Nucleolin	1.5372	0.0077968	nucleus
P31000	VIM	Vimentin	1.5458	0.048681	cytoplasm
P18437	HMGN2	Non-histone chromosomal protein HMG-17	1.5655	0.0025656	nucleus
A7VJC2	HNRNPA2B1	Heterogeneous nuclear ribonucleoproteins A2/B1	1.5758	0.021078	nucleus
Q5XIT1	MAPRE3	Microtubule-associated protein RP/EB family member 3	1.6249	0.00067394	cytoplasm
D3ZQL7	TPPP	Tubulin polymerization-promoting protein	1.632	0.00059322	cytoplasm
O08838	AMPH	Amphiphysin	1.6327	0.00036505	plasma membrane
Q9QX74	SHANK2	SH3 and multiple ankyrin repeat domains protein 2	1.634	0.029511	plasma membrane
Q66HL2	CTTN	Src substrate cortactin	1.6532	0.0004992	plasma membrane
P0DJJ3	SGIP1	SH3-containing GRB2-like protein 3-interacting protein 1	1.6566	4.14E-05	cytoplasm
Q4FZY0	EFHD2	EF-hand domain-containing protein D2	1.6817	0.00035801	plasma membrane
Q9Z327	SYNPO	Synaptopodin	1.6861	0.007485	cytoplasm
D4A6L0	GPR158	Metabotropic glycine receptor	1.7334	0.016391	plasma membrane
P25886	RPL29	Large ribosomal subunit protein eL29	1.7514	0.035094	cytoplasm
P41777	NOLC2	Nucleolar and coiled-body phosphoprotein 1	1.7578	0.0049207	nucleus
O08678	MARK1	Serine/threonine-protein kinase MARK1	1.7732	5.89E-05	cytoplasm
P24587	AKAP5	A-kinase anchor protein 5	1.7814	0.0014999	cytoplasm
P0DP31	CALM3	Calmodulin-3	1.7902	0.042776	cytoplasm
Q05175	BASP1	Brain acid soluble protein 1	1.8123	0.018521	nucleus
Q5XI72	EIF4H	Eukaryotic translation initiation factor 4H	1.833	0.0057392	cytoplasm
P84087	CPLX2	Complexin-2	1.866	0.0048925	cytoplasm
Q5M7W5	MAP4	Microtubule-associated protein 4	1.8737	0.00011353	cytoplasm
Q5XIM5	CDV3	Protein CDV3 homolog	1.9157	0.0025812	cytoplasm
Q99MZ8	LASP1	LIM and SH3 domain protein 1	1.9801	3.53E-05	cytoplasm
P36201	CRIP2	Cysteine-rich protein 2	2.0114	0.008439	plasma membrane
Q63560	MAP6	Microtubule-associated protein 6	2.1031	1.95E-05	cytoplasm
Q4KM31	LIMD2	LIM domain-containing protein 2	2.1794	0.020482	other
Q4KLH5	AGFG1	Arf-GAP domain and FG repeat-containing protein 1	2.2514	0.00032442	nucleus
P19332	MAPT	Microtubule-associated protein tau	2.3099	5.73E-05	plasma membrane
P07936	GAP43	Neuromodulin	2.3281	0.041175	plasma membrane
D4A0X3	BTBD8	BTB/POZ domain-containing protein 8	2.3785	0.0012843	nucleus
Q6KC51	ABLIM2	Actin-binding LIM protein 2	2.433	0.0016506	cytoplasm
P15146	MAP2	Microtubule-associated protein 2	2.4571	0.0026239	plasma membrane
Q04940	NRGN	Neurogranin	4.6043	0.0019245	cytoplasm

**Table 2:** Downregulated proteins showing decreased abundance in susceptible animals compared to resilient animals.

Accession	Symbol	Entrez gene name	FC	*p*-value	Location
P19527	NEFL	Neurofilament light polypeptide	0.46811	0.040581	cytoplasm
Q5RJQ4	SIRT2	NAD-dependent protein deacetylase sirtuin-2	0.49354	0.039494	nucleus
P43425	GNG7	Guanine nucleotide-binding protein G(I)/G(S)/G(O) subunit gamma-7	0.52585	0.013556	plasma membrane
Q6P7A2	UBE4A	Ubiquitin conjugation factor E4 A	0.52866	0.019417	cytoplasm
P23565	INA	Alpha-internexin	0.53671	0.047971	cytoplasm
D3ZPX4	PLXNA3	Plexin-A3	0.5449	0.0013272	plasma membrane
Q6VBQ5	MYADM	Myeloid-associated differentiation marker	0.59352	0.016709	nucleus
P61227	RAP2B	Ras-related protein Rap-2b	0.6017	0.0040688	plasma membrane
P11506	ATP2B2	Plasma membrane calcium-transporting ATPase 2	0.60772	0.024518	plasma membrane
P40241	CD9	CD9 antigen	0.61799	0.049211	plasma membrane
Q62745	CD81	CD81 antigen	0.63966	0.025148	plasma membrane
P36970	GPX4	Phospholipid hydroperoxide glutathione peroxidase	0.64734	0.021801	cytoplasm
P31647	SLC6A11	Sodium- and chloride-dependent GABA transporter 3	0.64748	0.047594	plasma membrane
P30713	GSTT2	Glutathione S-transferase theta-2	0.64785	0.0074908	cytoplasm
Q64542	ATP2B4	Plasma membrane calcium-transporting ATPase 4	0.64972	0.033987	plasma membrane

A volcano plot illustrates the variation in protein expression between susceptible and resilient rats (Figure 2B). These quantitative differences suggest that distinct proteins vary between the two groups, potentially linked to the behavioral resilience or susceptibility observed. In addition, hierarchical clustering shows patterns of similarity among the differentially expressed proteins across our experimental groups (Figure 2C). In the heatmap, the x-axis represents individual samples from the stress-resilient (*n* = 4) and stress-susceptible (*n* = 4) groups, while the y-axis lists the 53 differentially expressed proteins. Color intensity indicates relative protein abundance, with orange indicating higher and blue indicating lower expression. The analysis showed distinct clustering patterns, with the stress-susceptible group clustering separately from the stress-resilient group. This separation shows the unique protein expression profiles of each group. These findings confirm group-specific protein expression patterns and further support the distinct molecular profiles identified in the PCA analysis.

### 3.3 Identification of key biological pathways implicated in stress susceptibility

To identify the biological pathways associated with the differentially expressed proteins, we performed an Ingenuity Pathway Analysis (IPA). The analysis showed five enriched pathways, suggesting potential mechanisms underlying stress susceptibility (Figure 2D). Among the top pathways identified, clathrin-mediated endocytosis showed the highest activation score (Z-score = 2.236, *p* = 0.00000588), with key proteins involved, including AGFG1, AMPH, CTTN, SGIP1, and SH3KBP1 (Supplementary Figure 3). This pathway is critical for synaptic vesicle trafficking and receptor internalization, both essential for neuronal communication (Hanley, 2018; Prichard et al., 2022). The synaptogenesis signaling pathway was also significantly enriched (Z-score = 1, *p* = 0.00041), indicating potential alterations in neuronal connectivity (Petanjek et al., 2023). Key proteins implicated in this pathway included CALM3, CPLX2, MAPT, RAP2B, and SNCA (Supplementary Figure 4).

In contrast, the 14–3-3-mediated signaling pathway (Z-score = −1, *p* = 0.038) exhibited decreased predicted activity, suggesting potential impairments in cell growth, survival, apoptosis, and regulation of cytokines (Obsilova and Obsil, 2022). Among the dysregulated proteins in susceptible animals, MAPT, RAP2B, SNCA, and VIM were identified within this pathway (Supplementary Figure 5). Similarly, chaperone-mediated autophagy (Z-score = −1, *p* = 0.00014) showed a negative activation score, suggesting reduced activity of this lysosome-dependent process responsible for the degradation of certain intracellular components that is needed for cellular homeostasis (Kaushik and Cuervo, 2018). Dysregulated proteins associated with this pathway included EIF4H, GPX4, MAPT, and SNCA (Supplementary Figure 6). Finally, the calcium signaling pathway (Z-score = −2, *p* = 0.0000741) showed a significant downregulation, suggesting a disruption in intracellular calcium regulation. Key proteins, including AKAP5, ATP2B2, ATP2B4, CALM3, and RAP2B (Supplementary Figure 7), play important roles in calcium homeostasis (Di et al., 2015; Khariv et al., 2018; Prada et al., 2020; Tokumitsu and Sakagami, 2022).

### 3.4 Neurogranin and MAPT show similar expression by immunohistochemistry

Among the 53 differentially expressed proteins identified in the IL of stress-susceptible versus resilient female rats, MAPT and neurogranin emerged as particularly compelling candidates for further investigation. Both were significantly upregulated in susceptible animals and are known to play critical roles in synaptic plasticity and memory processes. MAPT contributes to microtubule stabilization and axonal transport, and its dysregulation has been linked to impaired synaptic function and neurodegenerative conditions (Zhang et al., 2016). Neurogranin, a postsynaptic protein enriched in dendritic spines, modulates calmodulin availability and is essential for calcium-dependent signaling pathways that underlie learning and memory (Zhong et al., 2009).

**Figure 3:**
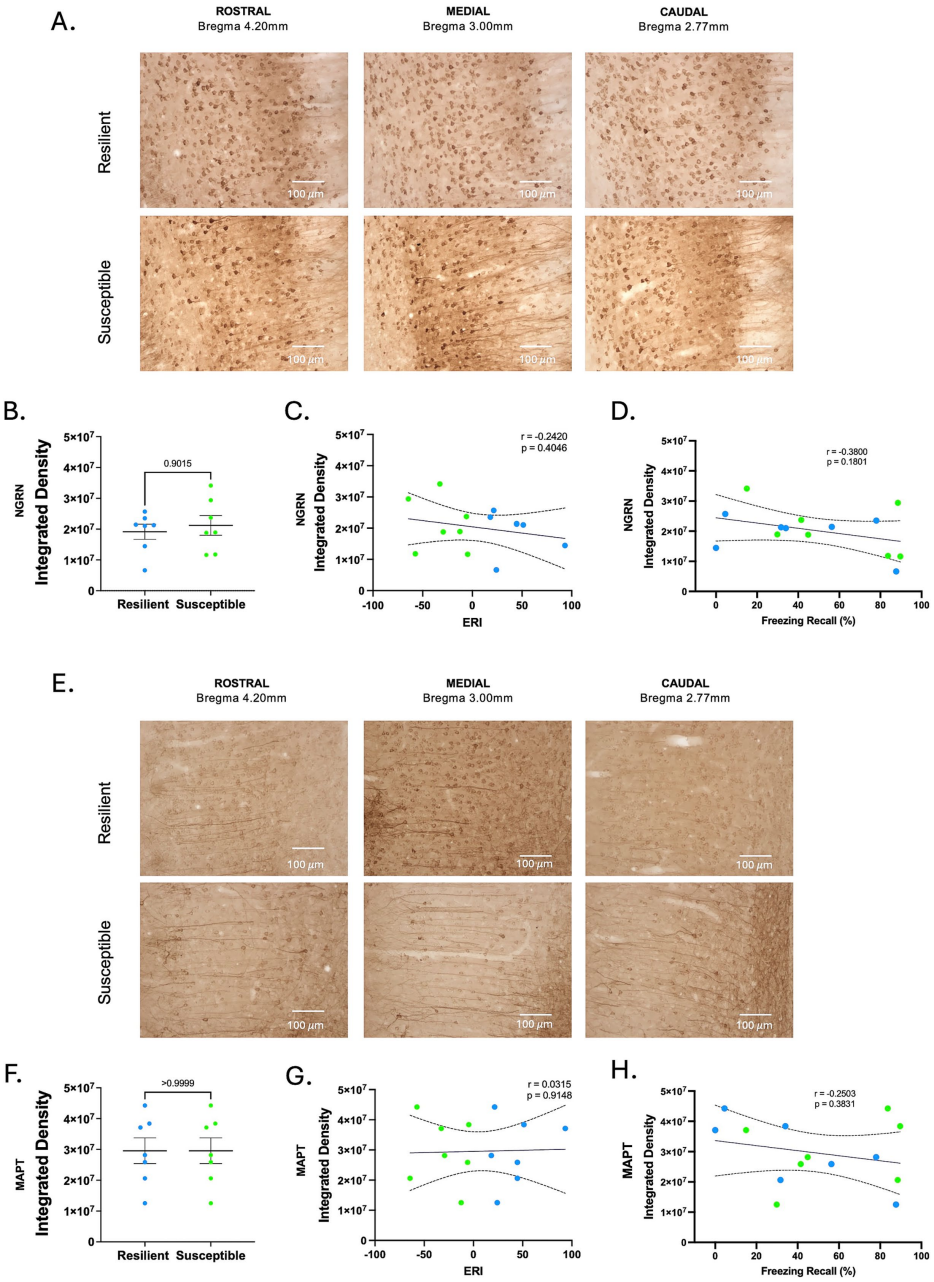
Immunohistological analysis of neurogranin and MAPT expression in IL. **(A)** Representative immunohistological sections stained for neurogranin in rostral, medial, and caudal regions (30 μm). **(B)** Comparison of neurogranin integrated density between groups. Unpaired *t*-test, *p* < 0.05. **(C)** Scatter plot showing the relationship between neurogranin integrated density and ERI. **(D)** Scatter plot showing the relationship between neurogranin integrated density and freezing percentage at recall. **(E)** Representative immunohistological sections stained for MAPT in rostral, medial, and caudal regions (30 m). **(F)** Comparison of MAPT integrated density between groups. Unpaired *t*-test, *p* < 0.05. **(G)** Scatter plot showing the relationship between MAPT integrated density and ERI. Data points represent individual animals. **(H)** Scatter plot showing the relationship between MAPT integrated density and freezing percentage at recall (Resilient, *n* = 7 vs. Susceptible, *n* = 7).

To assess whether the proteomic differences observed for neurogranin and MAPT were also detectable with an independent histological approach, we performed immunohistochemistry in IL of a separate cohort of resilient and susceptible female rats (Supplementary Figure 8). DAB-stained sections were semi-quantitatively analyzed using Image J by measuring the integrated density of positively stained areas (Figures 3A,E). Consistent with the literature showing expression of neurogranin and MAPT in pyramidal neurons (Watson et al., 1992; Singec et al., 2004; Karlsson et al., 2021; Vallés-Saiz et al., 2022), we observed abundant labeling of apical dendrites in the IL tissue. As shown in Figure 3B, no significant differences were observed in neurogranin expression in the IL between resilient and susceptible female rats (*p* = 0.9015). Furthermore, neurogranin integrated density values were not significantly correlated with ERI scores (*r* = − 0.2420, *p* = 0.4046; Figure 3C) or with freezing percentage at recall (*r* = − 0.3800, *p* = 0.1801, Figure 3D). Similarly, MAPT expression did not differ significantly between the two groups (*p* > 0.9999; Figure 3F), and no correlation was observed between MAPT integrated density with ERI scores (*r* = 0.0315, *p* = 0.9148; Figure 3G) or with freezing percentage at recall (*r* = −0.2503, *p* = 0.3831, Figure 3H). These findings indicate that, despite their upregulation in the proteomic dataset, neither neurogranin nor MAPT expression measured by immunohistochemistry in the IL was associated with the behavioral phenotype or extinction retention index in a separate cohort.

Although animals in this cohort were correctly classified as resilient or susceptible based on their ERI scores, their freezing levels during recall did not differ between groups (Supplementary Figure 8), potentially reducing the likelihood of detecting histological differences.

To further evaluate whether protein expression related to behavioral performance in this independent cohort, we stratified animals into lower and higher expression groups for neurogranin (Supplementary Figure 9A) and MAPT (Supplementary Figure 9D) using a median split. A two-way ANOVA showed no significant difference between lower and higher neurogranin expression groups (*F*(1, 12) = 0.03163, *p* = 0.8618) nor between lower and higher MAPT expression groups (*F*(1, 12) = 0.07369, *p* = 0.7907) (Supplementary Figures 9B,E) during fear conditioning or extinction. In addition, extinction recall did not differ between lower and higher neurogranin groups (*p* = 0.6690, Supplementary Figure 9C) or between lower and higher MAPT groups (*p* = 0.2768, Supplementary Figure 9F). Together, these results indicate that variability in neurogranin or MAPT expression was not associated with extinction recall performance in this cohort. Similarly, when animals were stratified by median split of extinction recall performance (Supplementary Figure 9G), neurogranin (*p* = 0.2011, Supplementary Figure 9H) and MAPT (*p* = 0.6715, Supplementary Figure 9I) integrated density values did not differ between groups. This pattern suggests that even with behavioral separation at extinction recall, we are unlikely to detect expression differences with immunohistochemistry.

### 3.5 Reducing neurogranin or MAPT expression in IL CAMKIIα-expressing neurons improves extinction memory following SPS

To further examine the role of neurogranin and MAPT in stress-induced extinction impairment, we tested the effects of reducing the expression of these proteins specifically in IL CAMKIIα-expressing neurons using viral delivery of shRNA constructs. Given the established roles of neurogranin and MAPT in neuronal signaling and plasticity, and their elevated expression in animals with impaired extinction memory, we hypothesized that reducing MAPT or neurogranin levels in IL neurons would improve extinction memory following traumatic stress. To test their causal involvement in stress-induced extinction impairment, we used AAV vectors expressing shRNA with a CAMKIIα promoter to selectively reduce these proteins in IL CAMKIIα-expressing neurons. Adult female rats received bilateral IL injections of AAV particles expressing shRNA targeting MAPT or neurogranin or control AAVs expressing a scramble-shRNA or channelrhodopsin. After a 30-day recovery period to allow viral expression, animals were exposed to the SPS protocol. One week later, rats were tested in the open field with a central object, followed by auditory fear conditioning and extinction. Only animals with confirmed IL infusions were included in behavioral and molecular analyses (Supplementary Figure 10).

To verify that the AAV shRNA constructs were expressed in CAMKIIα-expressing neurons, we quantified the percentage of virally infected cells (mCherry+) that co-expressed CAMKIIα within the IL. Across the three groups, the CAMKIIα promoter drove viral expression with consistently high specificity. The control group showed 88%, the shRNA NGRN exhibited 89% and shRNA MAPT displayed 91% colabeling between mCherry fluorescence and CAMKIIα immunofluorescence (Supplementary Figure 11). This indicates that approximately 80–90% of virally infected neurons expressed CAMKIIα, indicating high promoter specificity. A small proportion of mCherry+ neurons did not show detectable CAMKIIα labeling.

**Figure 4:**
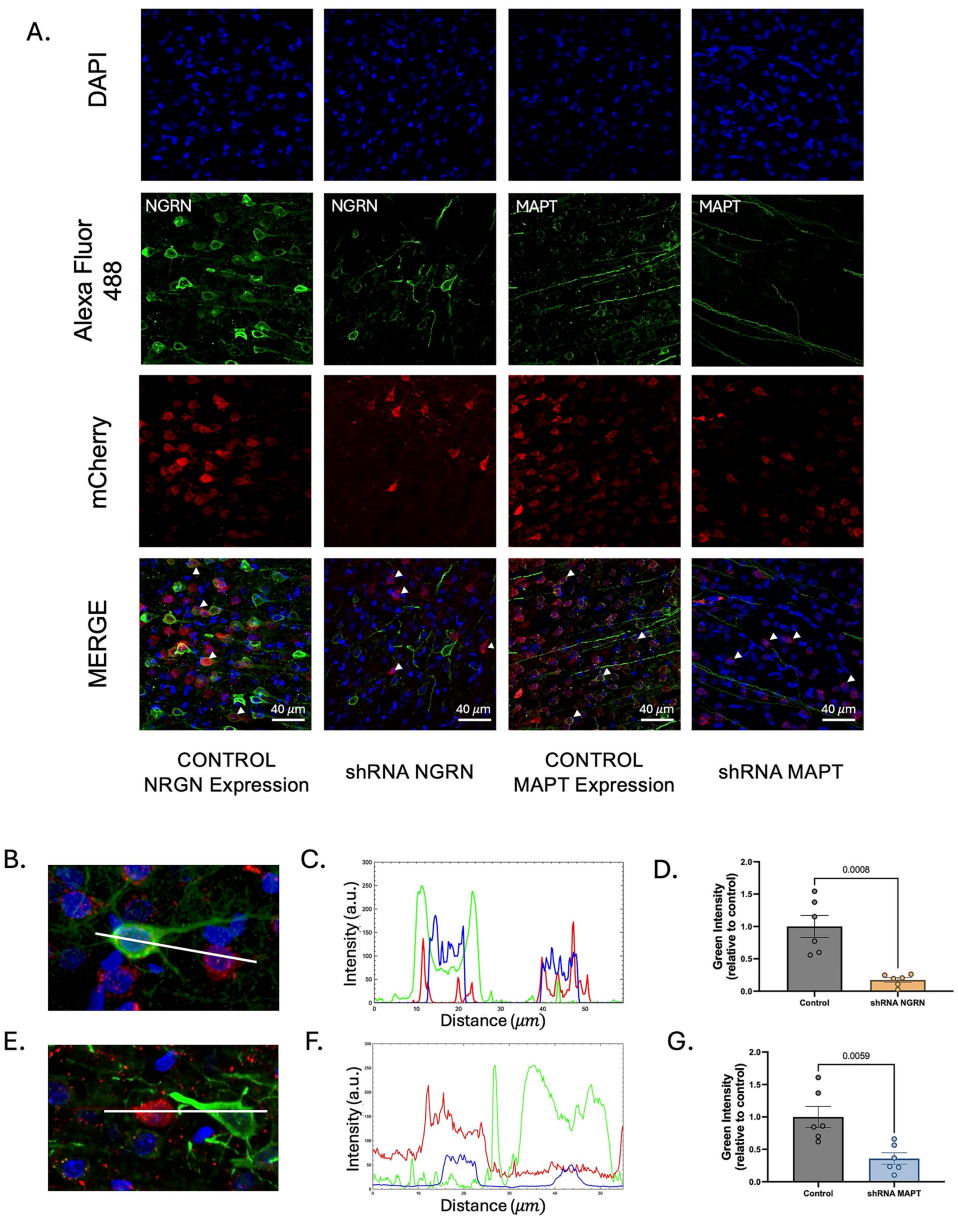
Validation of shRNA-mediated knockdown of neurogranin and MAPT in IL. **(A)** Representative images showing DAPI (blue), immunolabeled neurogranin or MAPT (green), and viral expression of mCherry (red) 1 month after infusion of AAV9 vectors. Arrowheads mark mCherry+ cells. Scale bar = 40 μm. **(B)** Example of an shRNA NGRN animal. Line scan used for fluorescence intensity profile of an infected neuron (mCherry^+^) and a non-infected (mCherry–) neighboring neuron to compare expression of neurogranin (green). **(C)** Representative intensity profile plot of raw fluorescence values for neurons shown in **B**. **(D)** Quantification of green intensity values of immunolabeled neurogranin in infected neurons from the shRNA NGRN group, normalized to infected neurons from the control group. Unpaired *t*-test, *p* < 0.05. **(E)** Example of an shRNA MAPT animal. Line scan used for fluorescence intensity profile of an infected neuron (mCherry^+^) compared to a non-infected (mCherry^−^) neighboring neuron to compare expression of MAPT (green). **(F)** Intensity profile plot of raw fluorescence values for neurons shown in **E**. **(G)** Quantification of normalized green intensity values of immunolabeled MAPT in infected neurons from the shRNA MAPT group, normalized to infected neurons from the control group. Unpaired *t*-test, *p* < 0.05. Each data point represents an individual cell.

To confirm shRNA efficacy, immunofluorescence staining was performed on IL sections collected after behavioral testing. Brain slices were labeled with MAPT- or neurogranin-specific antibodies, and the fluorescence intensity was quantified using ImageJ (Figure 4A). Line scans and intensity plots showed reduced immunolabeled neurogranin or MAPT in infected mCherry^+^ neurons compared to non-infected neighboring cells. (Figures 4B,C,E,F). Quantification of neurogranin expression showed a robust knockdown in infected neurons from shRNA NGRN animals compared to controls (mean normalized intensity = 0.1735 vs. 1.00), representing an ~83% reduction (Unpaired *t*-test, *t*(10) = 4.753, *p* = 0.0008, Figure 4D). Similarly, MAPT expression was significantly reduced in infected neurons from shRNA MAPT animals compared to controls (mean normalized intensity = 0.3585 vs. 1.00), corresponding to an ~64% reduction (Unpaired *t*-test, *t*(10) = 3.482, *p* = 0.0059, Figure 4G). These findings confirm that viral shRNA infusion effectively reduced neurogranin and MAPT expression in infected IL neurons.

**Figure 5:**
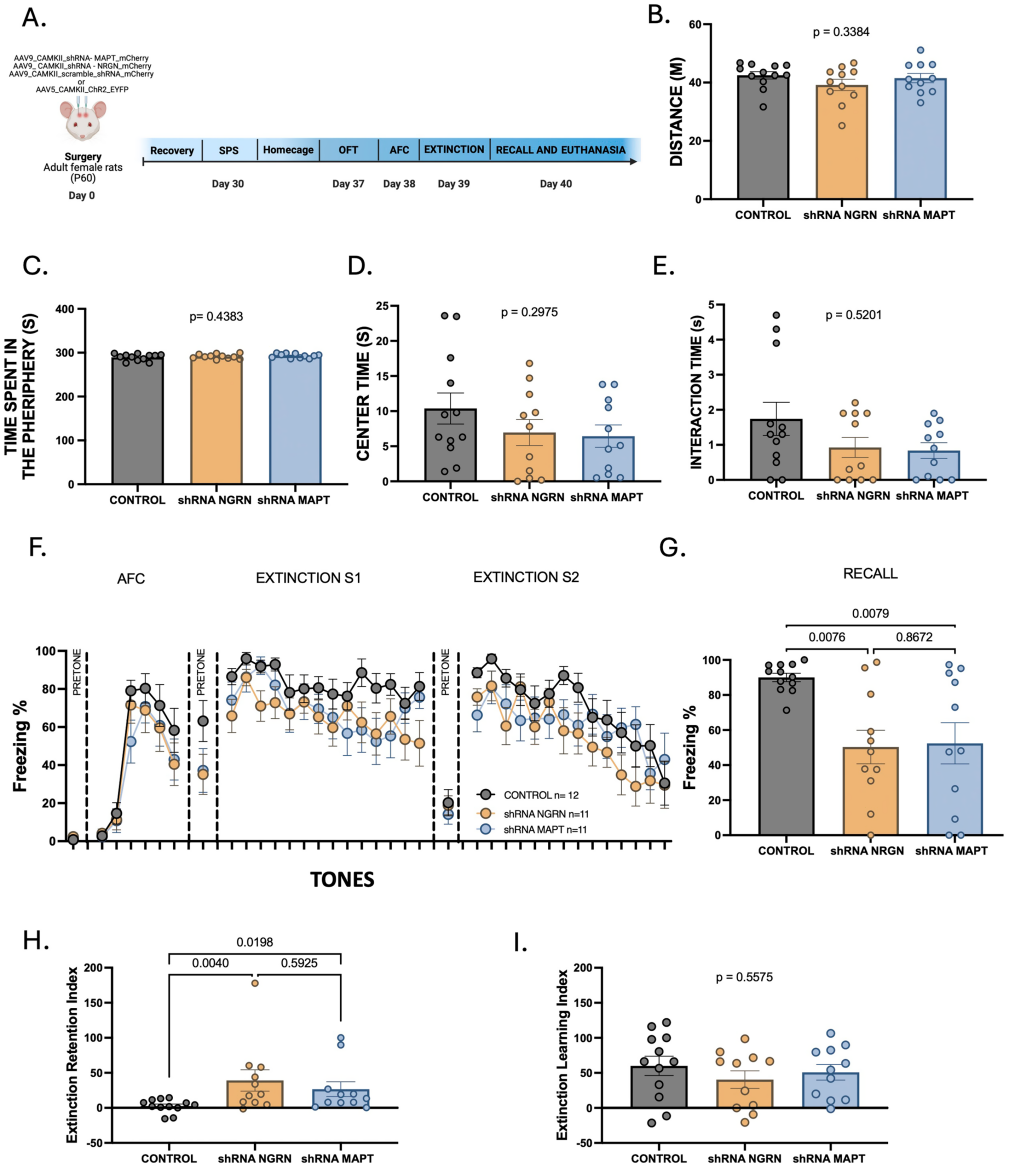
Reducing neurogranin or MAPT in IL improves extinction in SPS-exposed female rats. **(A)** Timeline for behavioral training and molecular assays in adult female rats infused with AAV expressing shRNA targeting neurogranin or MAPT or control AAV expressing a scramble shRNA or channelrhodopsin in the IL. CAMKII promoter targeted expression to glutamatergic neurons. **(B–E)** Comparison of exploratory behavior during the approach test among groups. **(F)** Freezing levels during auditory fear conditioning and extinction training in groups of females exposed to SPS. **(G)** Freezing levels during extinction recall among groups. One-way ANOVA, *p* < 0.05. **(H,I)** Comparison of ERI and ELI among groups. One-way ANOVA *p* < 0.05 Data points represent individual animals. Control *n* = 12, shRNA neurogranin *n* = 11, and shRNA MAPT *n* = 11. **(A)** was created with BioRender.com.

To assess whether knockdown of neurogranin or MAPT in IL neurons affects general locomotor or exploratory behavior, we conducted an open field test (Figure 5A). No significant differences were observed among groups in distance traveled [*F*(2, 31) = 1.122, *p* = 0.3384; Figure 5B], time spent in the periphery [*F*(2, 31) = 0.8472, *p* = 0.4383; Figure 5C], or center time [*F*(2, 31) = 1.261, *p* = 0.2975; Figure 5D]. Additionally, approach time toward the object did not differ significantly among groups [H (2) = 1.308, *p* = 0.5201; Figure 5E]. These findings indicate that IL knockdown of neurogranin or MAPT does not affect locomotor activity or exploratory drive.

We next examined whether IL knockdown of neurogranin or MAPT influences fear acquisition or extinction in adult female rats exposed to SPS (Figure 5A). During auditory fear conditioning in context A, baseline freezing during the pretone period, freezing during the first tone presentation, and freezing across the session did not differ among groups (Figure 5F). A two-way repeated measures ANOVA revealed that freezing behavior increased significantly across tone presentations [*F*(3.54, 109.7) = 55.05, *p* < 0.0001], indicating that all groups successfully acquired conditioned fear. However, there was no significant effect of treatment [*F*(2, 31) = 1.316, *p* = 0.2827] nor tone by treatment interaction [*F*(12, 186) = 0.6010, *p* = 0.8397]. Taken together, knockdown of neurogranin or MAPT in IL CAMKIIα-expressing neurons did not affect baseline contextual or auditory fear expression or acquisition of auditory conditioned fear, suggesting that the expression of these proteins in IL CAMKIIα-expressing neurons is not essential for encoding conditioned fear responses. On Day 2 (extinction training), rats were placed in Context B and exposed to two extinction sessions of 14 tones. A two-way repeated measures ANOVA revealed a significant effect of tone [*F*(9.14, 283.2) = 13.57, *p* < 0.0001], but no significant effect of treatment [*F*(2, 31) = 2.841, *p* = 0.0736] or tone by treatment interaction [*F*(58, 899) = 0.9633, *p* = 0.5548]. Freezing to the first two extinction tones, EXT1 and EXT2 (all *p* > 0.05) was similar across all groups. Freezing at the end of the extinction training was also comparable among groups (all *p* > 0.05), suggesting that knockdown of these proteins did not alter recall of the conditioned fear memory or extinction learning.

In contrast, during the extinction recall test (Day 3), IL knockdown of neurogranin or MAPT significantly reduced freezing compared to controls [*F*(2, 27) = 6.842, *p* = 0.0033]. *Post hoc* tests showed lower freezing in both shRNA-NRGN (*p* = 0.0070) and shRNA-MAPT (*p* = 0.0108) groups, with no difference between the knockdown groups (*p* = 0.9845; Figure 5G). Consistent with these behavioral effects, ERI scores were significantly lower in knockdown groups compared to control [H(2) = 9.452, *p* = 0.0089; Control vs. shRNA-NRGN, *p* = 0.0040; Control vs. shRNA-MAPT, *p* = 0.0198; Figure 5H], while ELI scores did not differ among groups [H(2) = 1.169, *p* = 0.5575; Figure 5I]. These results demonstrate that knockdown of neurogranin or MAPT in IL CAMKIIα-expressing neurons selectively enhances extinction memory following SPS exposure, without affecting baseline fear, auditory fear acquisition, or extinction learning in SPS-exposed female rats.

## 4 Discussion

### 4.1 Key findings

Impaired fear extinction is a hallmark of trauma-related disorders (Milad et al., 2009; Norrholm et al., 2011; Lokshina et al., 2023; Sep et al., 2023). Understanding the biological mechanisms that drive individual variability in extinction retention is critical in females, due to their underrepresentation in research. Such variability may reveal distinct vulnerability profiles in trauma-exposed individuals and guide the development of more effective interventions. Using the SPS animal model (Liberzon and Young, 1997; Liberzon et al., 1997), we identified distinct behavioral and proteomic phenotypes in female rats classified as stress-susceptible or resilient based on extinction retention performance. Quantitative proteomic analysis of the IL revealed distinct molecular profiles between these groups, with neurogranin and MAPT emerging as top candidates elevated in susceptible animals. Functional knockdown of either protein in IL CAMKIIα-expressing neurons enhanced extinction memory, establishing neurogranin and MAPT as molecular contributors to stress vulnerability in females and pointing to potential targets for extinction-based interventions.

The estrous cycle stage at recall did not significantly influence ERI scores, suggesting that hormonal fluctuations at the time of testing did not account for the variability. However, we cannot exclude the possibility that the estrous cycle stage during SPS exposure may contribute to stress susceptibility, as the literature has shown that estrous cycle stage and estradiol fluctuations can modulate both stress responses and extinction processes (Stockhorst and Antov, 2016; do Nascimento et al., 2019; Lovick and Zangrossi, 2021; Johnston et al., 2025).

### 4.2 Molecular insights into extinction impairment

Using TMT labeling, we identified 53 differentially expressed proteins, with 38 upregulated and 15 downregulated in the susceptible group. Principal component analysis confirmed distinct proteomic profiles between the two groups, reflecting stress-induced molecular changes in IL. These alterations may contribute to extinction retention deficits observed in susceptible animals, consistent with studies linking molecular changes in the fear circuitry to impaired extinction (Al Jowf et al., 2021). Pathway analysis implicated clathrin-mediated endocytosis, synaptogenesis signaling pathway, 14–3-3-mediated signaling, chaperone-mediated autophagy, and calcium signaling, processes that converge on mechanisms that regulate neuronal excitability, synaptic remodeling, and intracellular signaling (Kaushik and Cuervo, 2018; Obsilova and Obsil, 2022; Prichard et al., 2022; Tokumitsu and Sakagami, 2022; Petanjek et al., 2023). Dysregulation across these systems may reduce IL responsiveness, contributing to extinction deficits.

### 4.3 Methodological considerations in corroborating proteomic findings with immunohistochemistry

Neurogranin and MAPT emerged as compelling candidates due to their established roles in synaptic plasticity and memory (Zhong et al., 2009, 2015; Kimura et al., 2014; Zhang et al., 2016; Regan et al., 2021). While immunohistochemical analysis did not replicate the proteomic findings, this discrepancy may reflect both technical and biological factors. On the technical side, antibody-based detection has limited sensitivity and may fail to detect the specific peptide fragments or protein isoforms identified by mass spectrometry. Commercial monoclonal antibodies recognize a single epitope, which means they may not detect low-abundance isoforms or post-translational modifications. Discrepancies are common because mass spectrometry is more sensitive than methods such as ELISA or Western blot, and each technique targets different molecular features. Future studies can address this by testing alternative antibodies or generating specific antibodies against the exact peptide sequences identified in proteomics. On the biological side, although animals were correctly classified by their ERI, their freezing levels during recall did not differ substantially in this cohort, potentially reducing the likelihood of detecting histological differences (Supplementary Figure 8). Additional analyses support the interpretation that the absence of differences in neurogranin or MAPT expression likely reflects technical limitations of immunohistochemistry rather than a lack of behavioral divergence. When animals were stratified by neurogranin or MAPT protein expression, no differences in extinction recall were detected. Similarly, stratifying animals by freezing expression at extinction recall did not show differences in neurogranin or MAPT protein expression. Together, these results suggest that technical differences between immunohistochemistry and proteomic analysis led to differences in the findings.

As discussed by Hernández et al. (2014) and colleagues validation failures are often attributed to statistical overfitting or small sample sizes; however, the PCA clustering in our dataset suggests that our proteomic findings are not artifacts of overfitting but reflect meaningful molecular distinctions. These findings underscore the importance of integrating multiple modalities to fully characterize the molecular substrates of resilience and susceptibility and suggest that proteomic changes may precede or occur independently of detectable histological alterations. Although histological quantification did not reveal changes, the improvement in extinction recall following neurogranin or MAPT knockdown demonstrates their functional relevance to extinction memory.

### 4.4 Behavioral effects of shRNA are likely mediated by glutamatergic neurons in IL

The high percentage of infected cells that exhibited CAMKII immunolabeling (88%) suggests that the CAMKIIα promoter largely targets excitatory neurons in IL. However, a small fraction (~ 10%) of the infected cells did not show CAMKIIα labeling, which may reflect expression in GABAergic neurons. This is consistent with findings that certain cortical GABAergic neurons express CAMKIIα-driven viral constructs (Veres et al., 2023). Although GABAergic neurons may express the injected viral constructs, neurogranin and MAPT are largely expressed by glutamatergic neurons.

Neurogranin is highly enriched in cortical and hippocampal pyramidal neurons, supporting specificity to excitatory cell populations (Singec et al., 2004; Karlsson et al., 2021). MAPT, while more broadly expressed, is also most abundant in excitatory neurons, with lower expression levels in inhibitory and glial populations (Karlsson et al., 2021; Vallés-Saiz et al., 2022). Therefore, the enhanced extinction memory induced by reducing neurogranin and MAPT observed in our study likely reflects changes in glutamatergic neurons within IL, while not excluding potential minor contributions from GABAergic neurons.

### 4.5 Functional validation of neurogranin and MAPT in extinction deficits

Previous work showed that increasing neurogranin expression in the prefrontal cortex can enhance synaptic plasticity and facilitate extinction learning (Zhong et al., 2015), and that neurogranin regulates metaplasticity by shifting the threshold for long-term potentiation (LTP) and long-term depression (LTD) (Zhong et al., 2009). In contrast, our results suggest that stress-induced upregulation of neurogranin in IL may impair extinction. This discrepancy may be explained by differences in brain region, timing, and context of neurogranin upregulation. Although both IL and prelimbic (PL) cortices are part of the medial prefrontal cortex, they play opposing roles in fear regulation. IL promotes extinction, while PL promotes fear expression (Milad et al., 2009; Sierra-Mercado et al., 2011). Zhong and colleagues reported extinction facilitation using a transgenic model with developmentally regulated neurogranin overexpression in PL, whereas our findings reflect stress-induced, potentially dysregulated neurogranin elevation in the IL following SPS exposure. Excessive neurogranin may saturate calmodulin-dependent signaling pathways, occluding further plasticity required for extinction memory (Huang et al., 2004; Zhong et al., 2009). This aligns with the concept of metaplasticity, where prior synaptic activity alters the threshold for future plasticity (Abraham, 2008). Since SPS disrupts IL neuronal function, (Nawreen et al., 2021), elevated neurogranin after stress may reflect a maladaptive response that impaired the plasticity needed for extinction memory.

Our knockdown experiments targeting MAPT align with findings by Regan and colleagues (Regan et al., 2021), who showed that phosphorylation of tau at Ser396/404 disrupts its interaction with PACSIN1, leading to impaired AMPA receptor trafficking and synapse weakening. Although Regan did not examine extinction directly, these results suggest that tau dysregulation can compromise AMPA receptor–mediated transmission, a process essential for extinction. We found that reducing tau expression in IL pyramidal neurons improved extinction memory, suggesting that tau knockdown may restore AMPA receptor function and thereby rescue extinction-related plasticity. Kimura et al. demonstrated that tau is essential for LTD in the hippocampus, a form of plasticity involving AMPA receptor internalization that is critical for memory updating (Kimura et al., 2014). They showed that tau knockout or shRNA-mediated knockdown abolished LTD without affecting baseline synaptic transmission or LTP, and that LTD induction required GSK-3β–dependent phosphorylation of tau at Ser396/404. These findings suggest that tau dynamically regulates synaptic plasticity. Traumatic stress might dysregulate tau phosphorylation, shifting its role from adaptive to maladaptive and impairing extinction. Our results support that MAPT dysregulation in the IL following traumatic stress disrupts extinction-related plasticity, and that targeted reduction of tau expression can restore functional flexibility in fear circuits.

### 4.6 ERI as a measure of extinction retention

While freezing at recall is a commonly used metric, we consider ERI a more integrative measure of extinction. Unlike freezing at recall, which reflects only a single time point, ERI integrates both extinction learning and recall. This approach explains why some animals in the resilient group showed relatively high freezing on the recall day, and conversely, why some animals classified as susceptible showed low freezing. Thus, ERI captures whether extinction was retained relative to each animal’s learning, rather than relying on absolute freezing levels at recall. One limitation, however, is the potential misclassification of animals with atypical behavioral curves, such as those beginning extinction training with unusually low freezing despite normal fear conditioning, which may reflect impaired consolidation or weak expression of the fear memory. Animals failing to consolidate the fear should be excluded from extinction retention analyses.

### 4.7 Limitations and future directions

Several limitations should be acknowledged. First, the absence of a non-stress control group limits our ability to determine how SPS exposure alters the IL proteome. Instead, this study focuses on individual differences in susceptibility and resilience among stress-exposed animals, without providing a broader view of stress-induced changes relative to unstressed conditions. Future studies should incorporate non-stressed controls to provide a broader context for stress-induced changes. Second, our behavioral analysis focused solely on freezing behavior, which does not capture other fear-related responses such as darting, avoidance, or escape. Expanding behavioral assays to include these active coping strategies would provide a more comprehensive understanding of resilience and susceptibility.

Third, although we identified differentially expressed proteins, we did not assess their post-translational modifications, which are essential for regulating protein function and interactions. Investigating these modifications in future work will be critical for clarifying the functional significance of our proteomic findings. Moreover, proteomics does not reveal the cell-type specificity of the proteins identified, leaving open the question of which neuronal or glial populations and compartments contribute to the observed changes. Additionally, proteomic analysis may not effectively capture small proteins or peptides, potentially excluding key molecular players.

Our proteomic analysis employed a sample size commonly used in TMT-based workflows (Armina-Rodriguez et al., 2025; Morales-Cabán et al., 2025; Rivera-Serrano et al., 2025; Rosario-Rodríguez et al., 2025). Although a small sample size can limit the ability to capture the full range of biological variability, the clear separation observed in the PCA, and the robust statistical differences detected between susceptible and resilient groups indicate that the molecular signatures identified here are strong and not likely to be artifacts of underpowered sampling. Nevertheless, future studies with larger cohorts, along with complementary validation methods such as Western blotting or qPCR would be useful for further corroborating the observed protein differences.

Finally, the lack of evaluation of male rats limits the generalizability of our findings. Given the known sex differences in stress responses, neural plasticity, and extinction processes (Mancini et al., 2025), it remains unclear whether the molecular pathways identified here are specific to females or represent shared mechanisms across sexes. Future studies that include both male and female subjects are needed to determine sex dependent and sex independent features of stress susceptibility and resilience. Beyond these considerations, future studies should also explore the therapeutic potential of targeting MAPT and neurogranin in extinction-based interventions, which may offer promising strategies for treating trauma-related disorders.

## 5 Conclusion

Our study provides a proteomic characterization of the IL cortex in SPS-exposed female rats, revealing molecular signatures associated with stress-susceptibility and resilience. By integrating behavioral, proteomic, and functional approaches, our findings identify candidate pathways and proteins that may contribute to extinction deficits and offer potential targets for therapeutic intervention in trauma-related disorders in females.

## Data Availability

All the proteomics raw datasets generated in the current study have been deposited in the ProteomeXchange Consortium via the PRIDE a partner repository with a dataset identifier. Project accession: PXD069169. Project DOI: 10.6019/PXD069169.

## Footnotes

1
https://imagej.net/ij/download.html

2
https://en.vectorbuilder.com/vector/VB241016-1953kgu.html

3
https://en.vectorbuilder.com/vector/VB241104-1771kkp.html

4
https://en.vectorbuilder.com/vector/VB250116-1495puv.html

5
https://www.addgene.org/26969/

## References

[ref1] Abraham W. C. (2008). Metaplasticity: tuning synapses and networks for plasticity. Nat. Rev. Neurosci. 9, 387–387. doi: 10.1038/nrn2356, 18401345

[ref2] Al Jowf G. I. Snijders C. Rutten B. P. F. de Nijs L. Eijssen L. M. T. (2021). The molecular biology of susceptibility to post-traumatic stress disorder: highlights of epigenetics and epigenomics. Int. J. Mol. Sci. 22:10743. doi: 10.3390/ijms221910743, 34639084 PMC8509551

[ref3] Armina-Rodriguez A. Valdés Fernandez B. N. Ocasio-Malavé C. Cantres Rosario Y. M. Carrasquillo Carrión K. Meléndez L. M. . (2025). Quantitative proteomics reveals Fh15 as an antagonist of TLR4 downregulating the activation of NF-κB, inducible nitric oxide, phagosome Signaling pathways, and oxidative stress of LPS-stimulated macrophages. Int. J. Mol. Sci. 26:6914. doi: 10.3390/ijms26146914, 40725160 PMC12294962

[ref4] Beery A. K. Zucker I. (2011). Sex bias in neuroscience and biomedical research. Neurosci. Biobehav. Rev. 35, 565–572. doi: 10.1016/j.neubiorev.2010.07.002, 20620164 PMC3008499

[ref5] Benjet C. Bromet E. Karam E. G. Kessler R. C. McLaughlin K. A. Ruscio A. M. . (2016). The epidemiology of traumatic event exposure worldwide: results from the world mental health survey consortium. Psychol. Med. 46, 327–343. doi: 10.1017/S0033291715001981, 26511595 PMC4869975

[ref6] Collins B. Biddle M. Wood D. R. Boyapati S. Barth M. Jeun C. . (2023). The role of avoidance in modulating single prolonged stress effects on emotional memory in male and female rats. Behav. Brain Res. 452:114579. doi: 10.1016/j.bbr.2023.114579, 37451551 PMC10530017

[ref7] Cookson M. R. (2019). Proteomics: techniques and applications in neuroscience. J. Neurochem. 151, 394–396. doi: 10.1111/jnc.14867, 31625157 PMC6856389

[ref8] Corcoran K. A. Quirk G. J. (2007). Recalling safety: cooperative functions of the ventromedial prefrontal cortex and the Hippocampus in extinction. CNS Spectr. 12, 200–206. doi: 10.1017/S1092852900020915, 17329980

[ref9] Costanzo M. Caterino M. Salvatori I. Manganelli V. Ferri A. Misasi R. . (2022). Proteome data of neuroblastoma cells overexpressing neuroglobin. Data Brief 41:107843. doi: 10.1016/j.dib.2022.107843, 35128003 PMC8800053

[ref10] Di J. Huang H. Qu D. Tang J. Cao W. Lu Z. . (2015). Rap2B promotes proliferation, migration and invasion of human breast cancer through calcium-related ERK1/2 signaling pathway. Sci. Rep. 5:12363. doi: 10.1038/srep12363, 26201295 PMC4512009

[ref11] do Nascimento E. B. Dierschnabel A. L. de Macêdo Meiros A. Suchecki D. Silva R. H. Ribeiro A. M. (2019). Memory impairment induced by different types of prolonged stress is dependent on the phase of the estrous cycle in female rats. Horm. Behav. 115:104563. doi: 10.1016/j.yhbeh.2019.104563 31377100

[ref12] Etkin A. Wager T. D. (2007). Functional neuroimaging of anxiety: a Meta-analysis of emotional processing in PTSD, social anxiety disorder, and specific phobia. Am. J. Psychiatry 164, 1476–1488. doi: 10.1176/appi.ajp.2007.07030504, 17898336 PMC3318959

[ref13] Ewald J. D. Zhou G. Lu Y. Kolic J. Ellis C. Johnson J. D. . (2024). Web-based multi-omics integration using the analyst software suite. Nat. Protoc. 19, 1467–1497. doi: 10.1038/s41596-023-00950-4, 38355833

[ref14] Hanley J. G. (2018). The regulation of AMPA receptor endocytosis by dynamic protein-protein interactions. Front. Cell. Neurosci. 12:362. doi: 10.3389/fncel.2018.00362, 30364226 PMC6193100

[ref15] Hernández B. Parnell A. Pennington S. R. (2014). Why have so few proteomic biomarkers “survived” validation? (sample size and independent validation considerations). Proteomics 14, 1587–1592. doi: 10.1002/pmic.201300377, 24737731

[ref16] Hu J. Feng B. Zhu Y. Wang W. Xie J. Zheng X. (2017). “Gender differences in PTSD: susceptibility and resilience” in Gender differences in different contexts. ed. Zheng X. (London: IntechOpen).

[ref17] Huang K.-P. Huang F. L. Jäger T. Li J. Reymann K. G. Balschun D. (2004). Neurogranin/RC3 enhances long-term potentiation and learning by promoting calcium-mediated Signaling. J. Neurosci. 24, 10660–10669. doi: 10.1523/JNEUROSCI.2213-04.2004, 15564582 PMC6730132

[ref18] Hunter J. D. (2007). Matplotlib: a 2D graphics environment. Comput. Sci. Eng. 9, 90–95. doi: 10.1109/MCSE.2007.55

[ref19] Johnston M. P. Garcia-Castañeda B. I. Cedillo L. G. Patel S. K. Vargas V. S. Wanat M. J. (2025). Estrous cycle stage gates the effect of stress on reward learning. Neuropsychopharmacology 16:9. doi: 10.1038/s41386-025-02170-8, 40670622 PMC12824353

[ref20] Karlsson M. Zhang C. Méar L. Zhong W. Digre A. Katona B. . (2021). A single–cell type transcriptomics map of human tissues. Sci. Adv. 7:169. doi: 10.1126/sciadv.abh2169, 34321199 PMC8318366

[ref21] Kaushik S. Cuervo A. M. (2018). The coming of age of chaperone-mediated autophagy. Nat. Rev. Mol. Cell Biol. 19, 365–381. doi: 10.1038/s41580-018-0001-6, 29626215 PMC6399518

[ref22] Keller S. M. Schreiber W. B. Staib J. M. Knox D. (2015). Sex differences in the single prolonged stress model. Behav. Brain Res. 286, 29–32. doi: 10.1016/j.bbr.2015.02.034, 25721741 PMC5745062

[ref23] Khariv V. Acioglu C. Ni L. Ratnayake A. Li L. Tao Y.-X. . (2018). A link between plasma membrane calcium ATPase 2 (PMCA2), estrogen and estrogen receptor α signaling in mechanical pain. Sci. Rep. 8:17260. doi: 10.1038/s41598-018-35263-0, 30467368 PMC6250714

[ref24] Kimura T. Whitcomb D. J. Jo J. Regan P. Piers T. Heo S. . (2014). Microtubule-associated protein tau is essential for long-term depression in the hippocampus. Philos. Trans. R Soc. Lond. B Biol. Sci. 369:20130144. doi: 10.1098/rstb.2013.0144, 24298146 PMC3843876

[ref25] Knox D. George S. A. Fitzpatrick C. J. Rabinak C. A. Maren S. Liberzon I. (2012). Single prolonged stress disrupts retention of extinguished fear in rats. Learn. Mem. 19, 43–49. doi: 10.1101/lm.024356.111, 22240323 PMC3262971

[ref26] Koenen K. C. Ratanatharathorn A. Ng L. McLaughlin K. A. Bromet E. J. Stein D. J. . (2017). Posttraumatic stress disorder in the world mental health surveys. Psychol. Med. 47, 2260–2274. doi: 10.1017/S0033291717000708, 28385165 PMC6034513

[ref27] Lancaster C. L. Teeters J. B. Gros D. F. Back S. E. (2016). Posttraumatic stress disorder: overview of evidence-based assessment and treatment. J. Clin. Med. 5:105. doi: 10.3390/jcm5110105, 27879650 PMC5126802

[ref28] Liberzon I. Krstov M. Young E. A. (1997). Stress-restress: effects on ACTH and fast feedback. Psychoneuroendocrinology 22, 443–453. doi: 10.1016/S0306-4530(97)00044-9, 9364622

[ref29] Liberzon I. Young E. A. (1997). Effects of stress and glucocorticoids on CNS oxytocin receptor binding. Psychoneuroendocrinology 22, 411–422. doi: 10.1016/S0306-4530(97)00045-0, 9364620

[ref30] Likhtik E. (2005). Prefrontal control of the amygdala. J. Neurosci. 25, 7429–7437. doi: 10.1523/JNEUROSCI.2314-05.2005, 16093394 PMC6725290

[ref31] Lokshina Y. Sheynin J. Vogt G. S. Liberzon I. (2023). Fear extinction learning in posttraumatic stress disorder. Curr. Top. Behav. Neurosci. 64, 257–270. doi: 10.1007/7854_2023_436 37535308

[ref32] Lonsdorf T. B. Merz C. J. Fullana M. A. (2019). Fear extinction retention: is it what we think it is? Biol. Psychiatry 85, 1074–1082. doi: 10.1016/j.biopsych.2019.02.011, 31005240

[ref33] Lovick T. A. Zangrossi H. (2021). Effect of estrous cycle on behavior of females in rodent tests of anxiety. Front. Psych. 12:11065. doi: 10.3389/fpsyt.2021.711065, 34531768 PMC8438218

[ref34] Mancini G. F. Marchetta E. Riccardi E. Trezza V. Morena M. Campolongo P. (2021). Sex-divergent long-term effects of single prolonged stress in adult rats. Behav. Brain Res. 401:113096. doi: 10.1016/j.bbr.2020.113096, 33359571

[ref35] Mancini G. F. Torrisi S. A. Viho E. M. G. Meijer O. C. Leggio G. M. Campolongo P. (2025). Interindividual and sex differences in resilience and vulnerability to post-traumatic stress disorder (PTSD): insights from animal models. Biol. Sex Differ. 16:50. doi: 10.1186/s13293-025-00732-5, 40598622 PMC12219389

[ref36] Maren S. Holmes A. (2016). Stress and fear extinction. Neuropsychopharmacology 41, 58–79. doi: 10.1038/npp.2015.180, 26105142 PMC4677122

[ref37] Milad M. R. Pitman R. K. Ellis C. B. Gold A. L. Shin L. M. Lasko N. B. . (2009). Neurobiological basis of failure to recall extinction memory in posttraumatic stress disorder. Biol. Psychiatry 66, 1075–1082. doi: 10.1016/j.biopsych.2009.06.026, 19748076 PMC2787650

[ref38] Morales-Cabán B. M. Cantres-Rosario Y. M. Tosado-Rodríguez E. L. Roche-Lima A. Meléndez L. M. Boukli N. M. . (2025). SCAMP3-driven regulation of ERK1/2 and autophagy Phosphoproteomics signatures in triple-negative breast Cancer. Int. J. Mol. Sci. 26:9577. doi: 10.3390/ijms26199577, 41096842 PMC12525412

[ref39] National Center for PTSD. (2018). How common is PTSD in adults? Available online at: https://www.ptsd.va.gov/understand/common/common_adults.asp (Accessed September 22, 2021).

[ref40] Nawreen N. Baccei M. L. Herman J. P. (2021). Single prolonged stress reduces intrinsic excitability and excitatory synaptic drive onto pyramidal neurons in the infralimbic prefrontal cortex of adult male rats. Front. Cell. Neurosci. 15:660. doi: 10.3389/fncel.2021.705660, 34366790 PMC8342808

[ref41] Neuner S. M. Wilmott L. A. Hope K. A. Hoffmann B. Chong J. A. Abramowitz J. . (2015). TRPC3 channels critically regulate hippocampal excitability and contextual fear memory. Behav. Brain Res. 281, 69–77. doi: 10.1016/j.bbr.2014.12.018, 25513972 PMC4677051

[ref42] Norrholm S. D. Jovanovic T. Olin I. W. Sands L. A. Karapanou I. Bradley B. . (2011). Fear extinction in traumatized civilians with posttraumatic stress disorder: relation to symptom severity. Biol. Psychiatry 69, 556–563. doi: 10.1016/j.biopsych.2010.09.013, 21035787 PMC3052965

[ref43] Obsilova V. Obsil T. (2022). Structural insights into the functional roles of 14-3-3 proteins. Front. Mol. Biosci. 9:1607. doi: 10.3389/fmolb.2022.1016071, 36188227 PMC9523730

[ref44] Olff M. (2017). Sex and gender differences in post-traumatic stress disorder: an update. Eur. J. Psychotraumatol. 8:1351204. doi: 10.1080/20008198.2017.1351204

[ref45] Pan H. Q. Liu X. X. He Y. Zhou J. Liao C. Z. You W. J. . (2022). Prefrontal GABAA(δ)R promotes fear extinction through enabling the plastic regulation of neuronal intrinsic excitability. J. Neurosci. 42, 5755–5770. doi: 10.1523/JNEUROSCI.0689-22.2022, 35705488 PMC9302468

[ref46] Pang Z. Lu Y. Zhou G. Hui F. Xu L. Viau C. . (2024). MetaboAnalyst 6.0: towards a unified platform for metabolomics data processing, analysis and interpretation. Nucleic Acids Res. 52, W398–W406. doi: 10.1093/nar/gkae253, 38587201 PMC11223798

[ref47] Petanjek Z. Banovac I. Sedmak D. Hladnik A. (2023). Dendritic spines: synaptogenesis and synaptic pruning for the developmental Organization of Brain Circuits. Adv. Neurobiol. 34, 143–221. doi: 10.1007/978-3-031-36159-3_4 37962796

[ref48] Piggott V. M. Bosse K. E. Lisieski M. J. Strader J. A. Stanley J. A. Conti A. C. . (2019). Single-prolonged stress impairs prefrontal cortex control of amygdala and striatum in rats. Front. Behav. Neurosci. 13:18. doi: 10.3389/fnbeh.2019.00018, 31114487 PMC6502983

[ref49] Pooley A. E. Benjamin R. C. Sreedhar S. Eagle A. L. Robison A. J. Mazei-Robison M. S. . (2018a). Sex differences in the traumatic stress response: PTSD symptoms in women recapitulated in female rats. Biol. Sex Differ. 9:31. doi: 10.1186/s13293-018-0191-9, 29976248 PMC6034295

[ref50] Pooley A. E. Benjamin R. C. Sreedhar S. Eagle A. L. Robison A. J. Mazei-Robison M. S. . (2018b). Sex differences in the traumatic stress response: the role of adult gonadal hormones. Biol. Sex Differ. 9:32. doi: 10.1186/s13293-018-0192-8, 30001741 PMC6043950

[ref51] Prada M. P. Syed A. U. Reddy G. R. Martín-Aragón Baudel M. Flores-Tamez V. A. Sasse K. C. . (2020). AKAP5 complex facilitates purinergic modulation of vascular L-type Ca2+ channel CaV1.2. Nat. Commun. 11:5303. doi: 10.1038/s41467-020-18947-y, 33082339 PMC7575592

[ref52] Prichard K. L. O’Brien N. S. Murcia S. R. Baker J. R. McCluskey A. (2022). Role of clathrin and dynamin in clathrin mediated endocytosis/synaptic vesicle recycling and implications in neurological diseases. Front. Cell. Neurosci. 15:754110. doi: 10.3389/fncel.2021.754110 35115907 PMC8805674

[ref53] Regan P. Mitchell S. J. Kim S. C. Lee Y. Yi J. H. Barbati S. A. . (2021). Regulation of synapse weakening through interactions of the microtubule associated protein tau with pacsin1. J. Neurosci. 41, 7162–7170. doi: 10.1523/JNEUROSCI.3129-20.2021, 34290082 PMC8387117

[ref54] Rivera-Serrano M. Flores-Colón M. Valiyeva F. Meléndez L. M. Vivas-Mejía P. E. (2025). Upregulation of MMP3 promotes cisplatin resistance in ovarian Cancer. Int. J. Mol. Sci. 26:4012. doi: 10.3390/ijms26094012, 40362252 PMC12071843

[ref55] Rosario-Rodríguez L. J. Cantres-Rosario Y. M. Rodríguez De Jesús A. E. Mera-Pérez A. M. Tosado-Rodríguez E. L. Roche Lima A. . (2025). Cannabinoid receptor type 2 agonist JWH-133 stimulates antiviral factors and decreases proviral, inflammatory, and neurotoxic proteins in HIV-infected macrophage secretome. Int. J. Mol. Sci. 26:10596. doi: 10.3390/ijms262110596, 41226631 PMC12608856

[ref56] Sep M. S. C. Geuze E. Joëls M. (2023). Impaired learning, memory, and extinction in posttraumatic stress disorder: translational meta-analysis of clinical and preclinical studies. Transl. Psychiatry 13:376. doi: 10.1038/s41398-023-02660-7, 38062029 PMC10703817

[ref57] Shansky R. M. Murphy A. Z. (2021). Considering sex as a biological variable will require a global shift in science culture. Nat. Neurosci. 24, 457–464. doi: 10.1038/s41593-021-00806-8, 33649507 PMC12900283

[ref58] Sierra-Mercado D. Corcoran K. A. Lebrón-Milad K. Quirk G. J. (2006). Inactivation of the ventromedial prefrontal cortex reduces expression of conditioned fear and impairs subsequent recall of extinction. Eur. J. Neurosci. 24, 1751–1758. doi: 10.1111/j.1460-9568.2006.05014.x, 17004939

[ref59] Sierra-Mercado D. Padilla-Coreano N. Quirk G. J. (2011). Dissociable roles of prelimbic and infralimbic cortices, ventral Hippocampus, and basolateral amygdala in the expression and extinction of conditioned fear. Neuropsychopharmacology 36, 529–538. doi: 10.1038/npp.2010.184, 20962768 PMC3005957

[ref60] Singec I. Knoth R. Ditter M. Volk B. Frotscher M. (2004). Neurogranin is expressed by principal cells but not interneurons in the rodent and monkey neocortex and hippocampus. J. Comp. Neurol. 479, 30–42. doi: 10.1002/cne.20302, 15389613

[ref61] Soler-Cedeño O. Torres-Rodríguez O. Bernard F. Maldonado L. Hernández A. Porter J. T. (2019). Plasticity of NMDA receptors at ventral hippocampal synapses in the infralimbic cortex regulates cued fear. eNeuro 6:ENEURO.0354-18.2019. doi: 10.1523/ENEURO.0354-18.2019, 30923737 PMC6437655

[ref62] Stockhorst U. Antov M. I. (2016). Modulation of fear extinction by stress, stress hormones and Estradiol: a review. Front. Behav. Neurosci. 9:359. doi: 10.3389/fnbeh.2015.00359, 26858616 PMC4726806

[ref63] Tokumitsu H. Sakagami H. (2022). Molecular mechanisms underlying Ca2+/calmodulin-dependent protein kinase kinase signal transduction. Int. J. Mol. Sci. 23:11025. doi: 10.3390/ijms231911025, 36232320 PMC9570080

[ref64] Torres-Rodriguez O. Ortiz-Nazario E. Rivera-Escobales Y. Velazquez B. Colón M. Porter J. T. (2023). Sex-dependent effects of microglial reduction on impaired fear extinction induced by single prolonged stress. Front. Behav. Neurosci. 16:14767. doi: 10.3389/fnbeh.2022.1014767, 36699653 PMC9868263

[ref65] Torres-Rodríguez O. Rivera-Escobales Y. Castillo-Ocampo Y. Velazquez B. Colón M. Porter J. T. (2023). Purinergic P2X7 receptor-mediated inflammation precedes PTSD-related behaviors in rats. Brain Behav. Immun. 110, 107–118. doi: 10.1016/j.bbi.2023.02.015, 36822379 PMC10106407

[ref66] Vallés-Saiz L. Ruiz-Gabarre D. García-Escudero V. Perry G. Avila J. Hernández F. (2022). Mouse and human tau expression in different brain areas. J. Alzheimers Dis. Rep. 6, 677–684. doi: 10.3233/ADR-220051, 36506485 PMC9696674

[ref67] Veres J. M. Andrasi T. Nagy-Pal P. Hajos N. (2023). CaMKIIα promoter-controlled circuit manipulations target both pyramidal cells and inhibitory interneurons in cortical networks. eNeuro 10:ENEURO.0070-23.2023. doi: 10.1523/ENEURO.0070-23.2023, 36963833 PMC10088982

[ref68] Waskom M. (2021). Seaborn: statistical data visualization. J. Open Source Softw. 6:3021. doi: 10.21105/joss.03021

[ref69] Watson J. B. Sutcliffe J. G. Fisher R. S. (1992). Localization of the protein kinase C phosphorylation/calmodulin-binding substrate RC3 in dendritic spines of neostriatal neurons. Proc. Natl. Acad. Sci. 89, 8581–8585. doi: 10.1073/pnas.89.18.8581, 1528865 PMC49964

[ref70] Zhang C.-C. Xing A. Tan M.-S. Tan L. Yu J.-T. (2016). The role of MAPT in neurodegenerative diseases: genetics, mechanisms and therapy. Mol. Neurobiol. 53, 4893–4904. doi: 10.1007/s12035-015-9415-8, 26363795

[ref71] Zhong L. Brown J. Kramer A. Kaleka K. Petersen A. Krueger J. N. . (2015). Increased prefrontal cortex neurogranin enhances plasticity and extinction learning. J. Neurosci. 35, 7503–7508. doi: 10.1523/JNEUROSCI.0274-15.2015, 25972176 PMC4429154

[ref72] Zhong L. Cherry T. Bies C. E. Florence M. A. Gerges N. Z. (2009). Neurogranin enhances synaptic strength through its interaction with calmodulin. EMBO J. 28, 3027–3039. doi: 10.1038/emboj.2009.236, 19713936 PMC2736013

