# Supplementary material for: Proteomic insights into extinction memory deficits in stress-susceptible female rats
Source: Front Behav Neurosci. 2026 Jan 13;19:1703714. doi: 10.3389/fnbeh.2025.1703714 (PMC12835298; doi:10.3389/fnbeh.2025.1703714)
Supplement: Supplementary file 2 [file Data_Sheet_1.PDF]

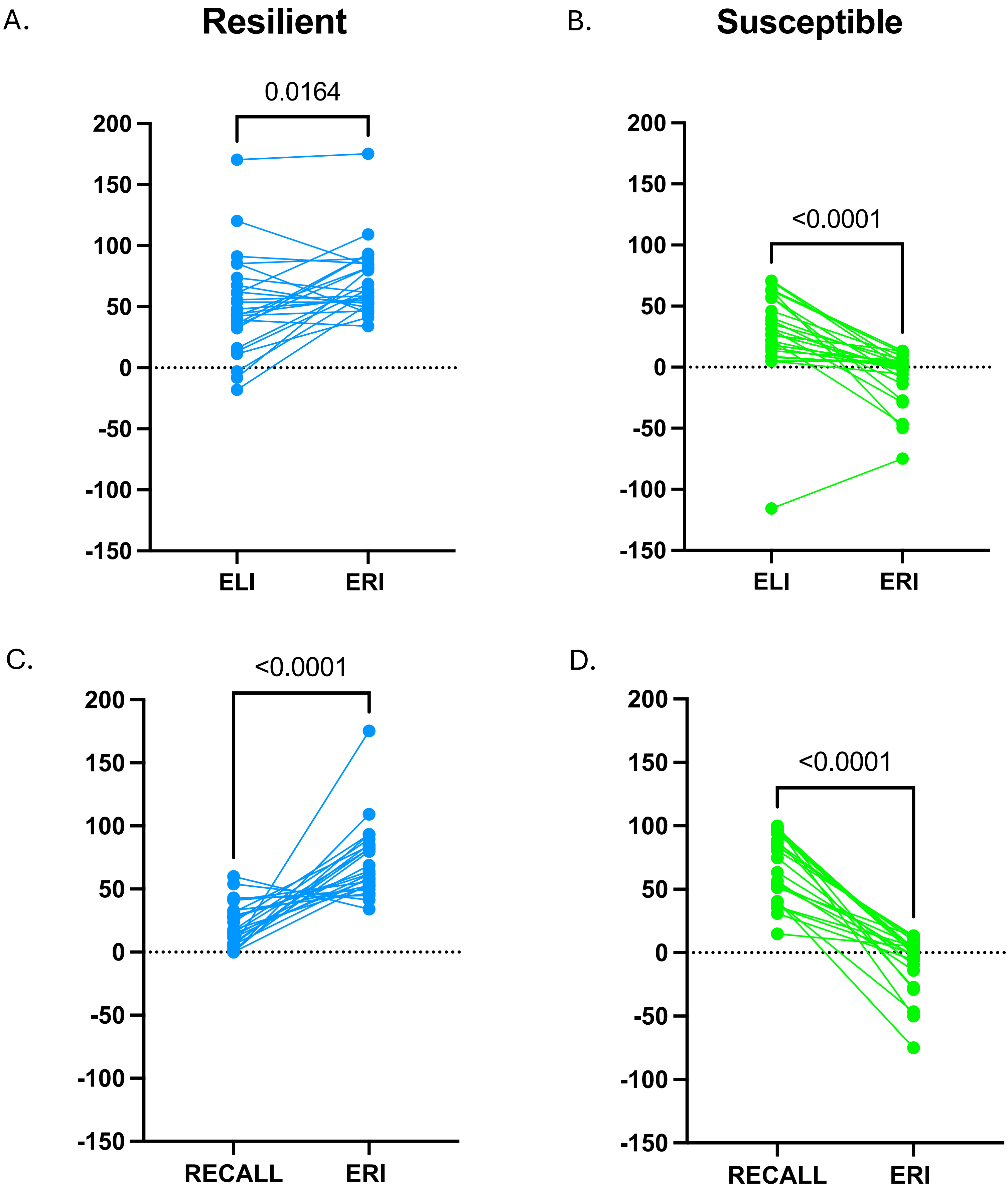

Supplementary figure 1 : Behavioral comparison of extinction learning, extinction retention and recall in susceptible and resilient animals. (A) Comparison between extinction learning and extinction retention in resilient animals. (B) Comparison between extinction learning and extinction retention in susceptible animals. (C) Comparison between recall and extinction retention in resilient animals. (D) Correlation between recall and extinction retention in susceptible animals. Data points represent individual animals susceptible n=20 and resilient n=18. Wilcoxon test, p-value <0.05

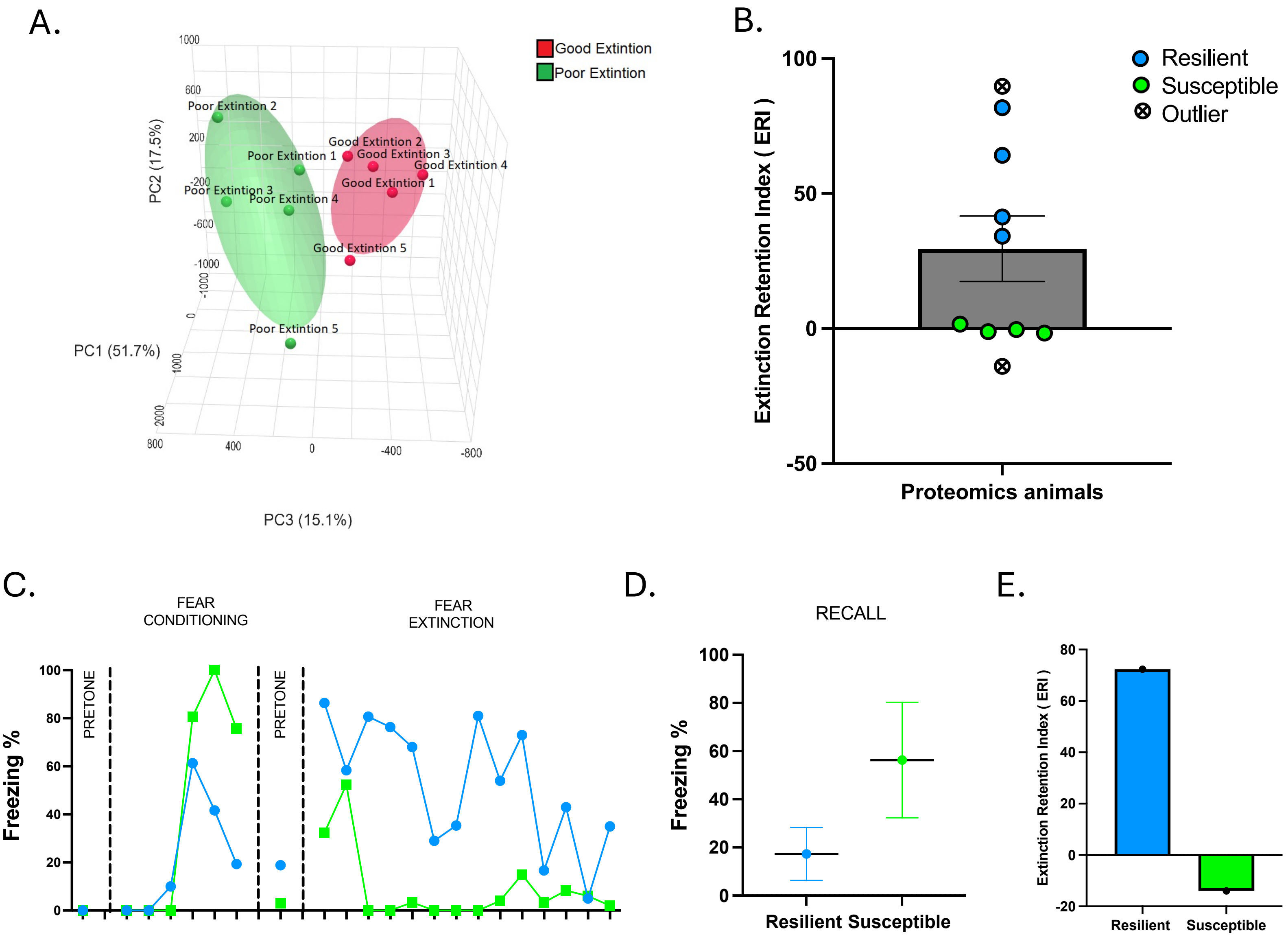

Supplementary figure 2: PCA score plot showed differences between stress-susceptible group, and stress-resilient group. PERMANOVA analysis F-value: 2.2397 ; R-squared: 0.21872 ; p-value (based on 999 permutations): p = 0.122. Each point represent individual animals susceptible-poor extinction (n=5) and resilient-good extinction (n=5). (B) Extinction Retention Index of animals used in proteomic experiments. Outliers are represented as a circle with an 'x'. (C) Freezing levels of auditory fear conditioning and extinction training of outliers animals . (D) Freezing levels during extinction recall of outlier animals. (E) ERI Scores of outliers.

Clathrin Mediated Endocytosis

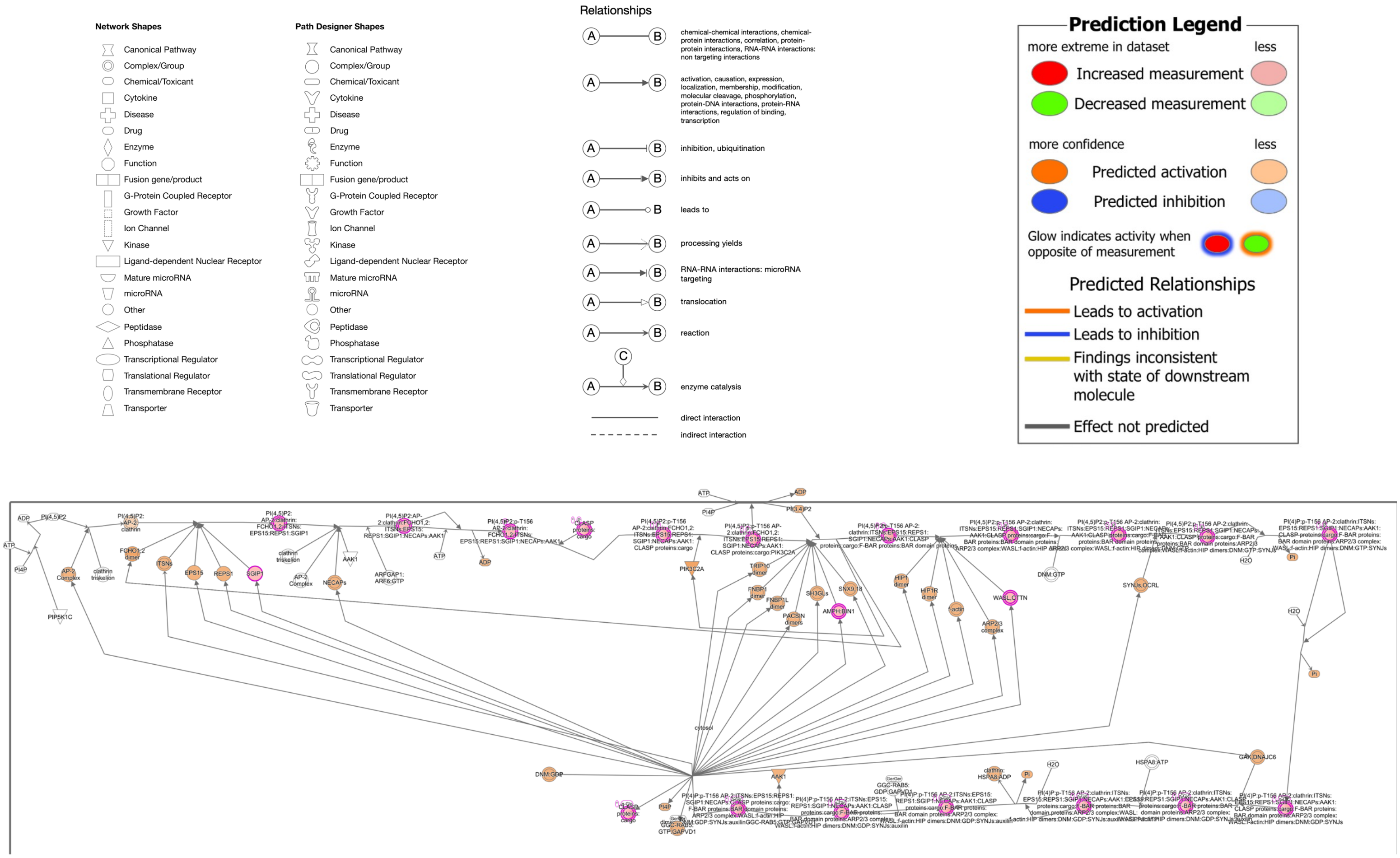

Supplementary Figure 3. Canonical Pathway of Clathrin Mediated Endocytosis of the susceptible females compared to the resilient females. This figure was obtained from IPA and integrates both measured expression changes and predicted molecular activity. Nodes are color-coded to reflect the direction and magnitude of expression changes, with red and pink indicating increased expression and green and light green indicating decreased expression, where darker shades represent more extreme values. Predicted activation and inhibition are represented by orange and blue nodes, respectively, with lighter shades indicating lower confidence. Glow effects around nodes denote activity opposite to the measured direction, suggesting potential compensatory mechanisms. Edges between nodes represent predicted molecular relationships: orange lines indicate activation, blue lines indicate inhibition, yellow lines highlight inconsistencies between predictions and downstream molecular states, and gray lines represent relationships with no predicted effect.

Synaptogenesis signaling pathway

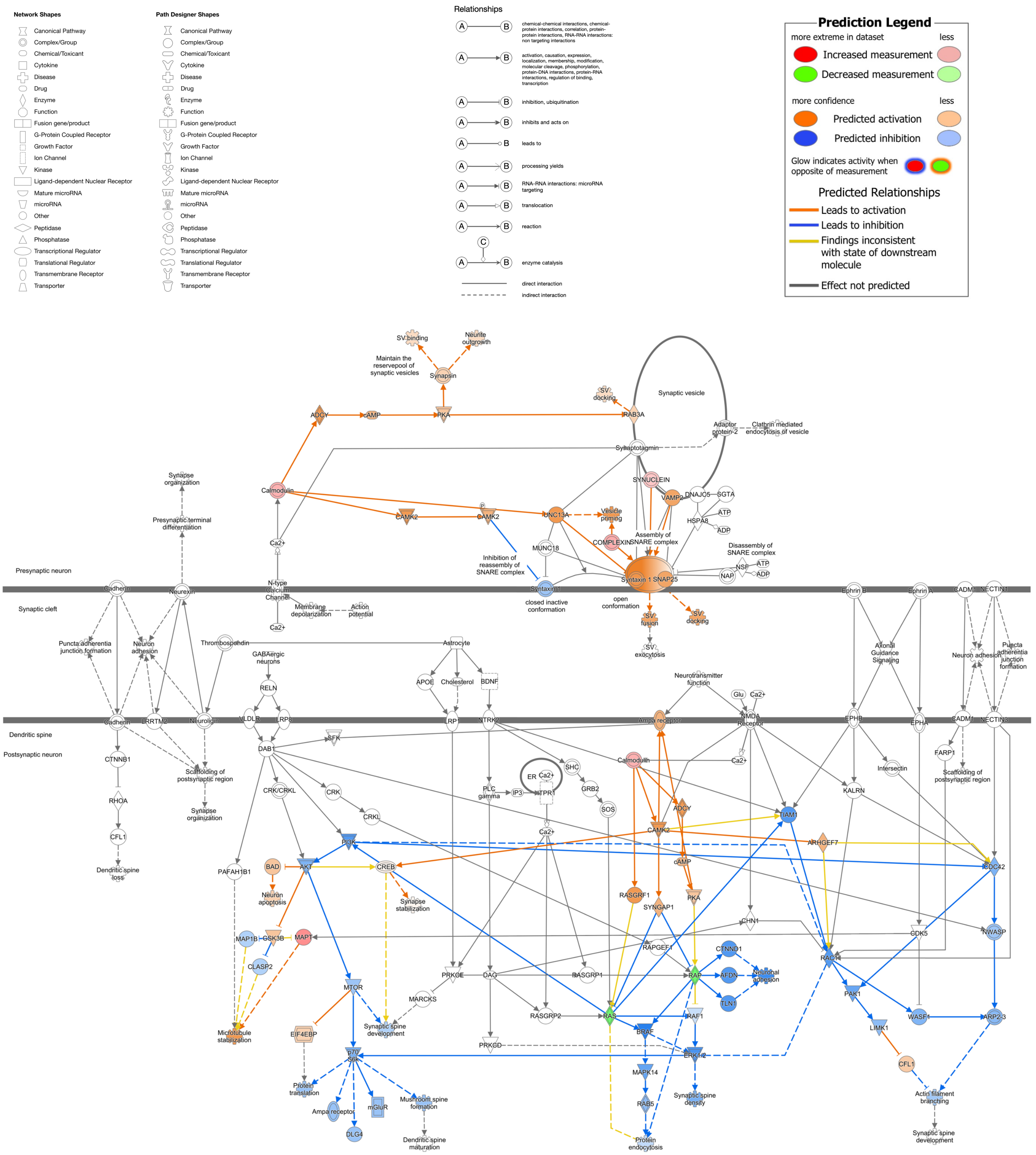

Supplementary Figure 4. Canonical Pathway of synaptogenesis of the susceptible females compared to the resilient females. This figure was obtained from IPA and integrates both measured expression changes and predicted molecular activity. Nodes are color-coded to reflect the direction and magnitude of expression changes, with red and pink indicating increased expression and green and light green indicating decreased expression, where darker shades represent more extreme values. Predicted activation and inhibition are represented by orange and blue nodes, respectively, with lighter shades indicating lower confidence. Glow effects around nodes denote activity opposite to the measured direction, suggesting potential compensatory mechanisms. Edges between nodes represent predicted molecular relationships: orange lines indicate activation, blue lines indicate inhibition, yellow lines highlight inconsistencies between predictions and downstream molecular states, and gray lines represent relationships with no predicted effect.

14-3-3 mediated signaling pathway

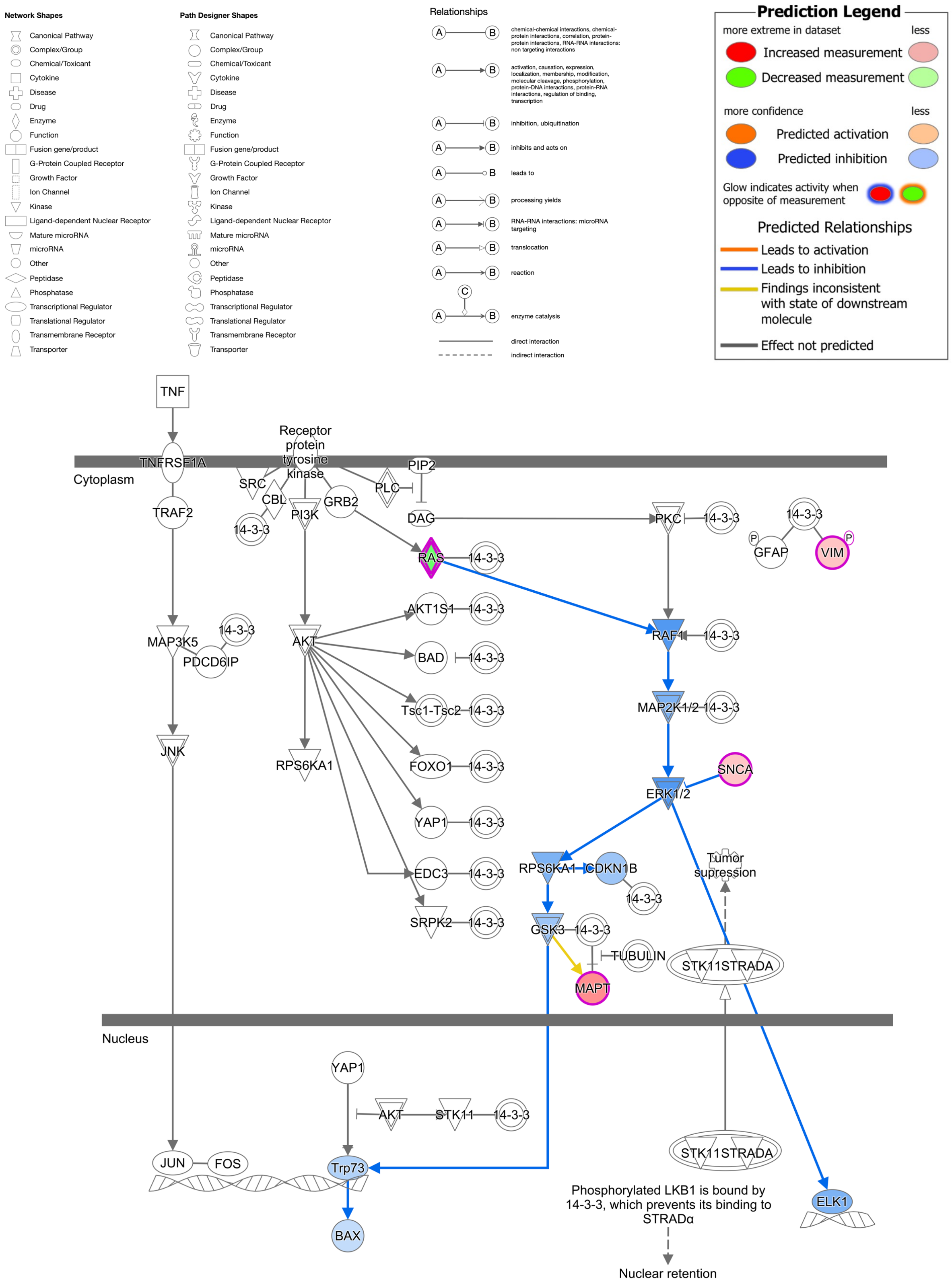

Supplementary Figure 5. Canonical Pathway of 14-3-3 mediated signaling of the susceptible females compared to the resilient females. This figure was obtained from IPA and integrates both measured expression changes and predicted molecular activity. Nodes are color-coded to reflect the direction and magnitude of expression changes, with red and pink indicating increased expression and green and light green indicating decreased expression, where darker shades represent more extreme values. Predicted activation and inhibition are represented by orange and blue nodes, respectively, with lighter shades indicating lower confidence. Glow effects around nodes denote activity opposite to the measured direction, suggesting potential compensatory mechanisms. Edges between nodes represent predicted molecular relationships: orange lines indicate activation, blue lines indicate inhibition, yellow lines highlight inconsistencies between predictions and downstream molecular states, and gray lines represent relationships with no predicted effect.

Chaperone mediated autophagy signaling pathway

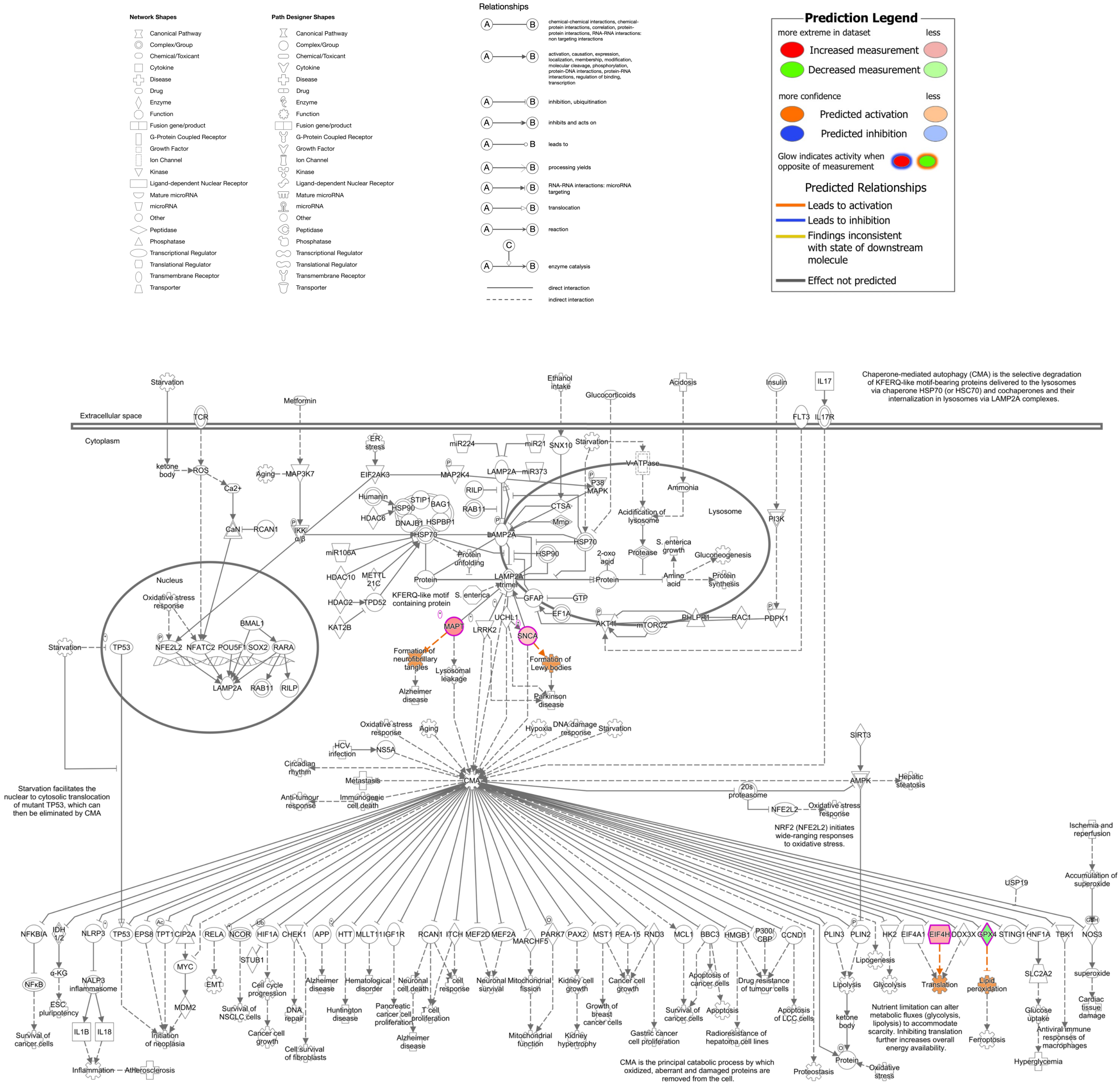

Supplementary Figure 6. Canonical Pathway of chaperone mediated autophagy signaling pathway of the susceptible females compared to the resilient females. This figure was obtained from IPA and integrates both measured expression changes and predicted molecular activity. Nodes are color-coded to reflect the direction and magnitude of expression changes, with red and pink indicating increased expression and green and light green indicating decreased expression, where darker shades represent more extreme values. Predicted activation and inhibition are represented by orange and blue nodes, respectively, with lighter shades indicating lower confidence. Glow effects around nodes denote activity opposite to the measured direction, suggesting potential compensatory mechanisms. Edges between nodes represent predicted molecular relationships: orange lines indicate activation, blue lines indicate inhibition, yellow lines highlight inconsistencies between predictions and downstream molecular states, and gray lines represent relationships with no predicted effect.

Calcium signaling pathway

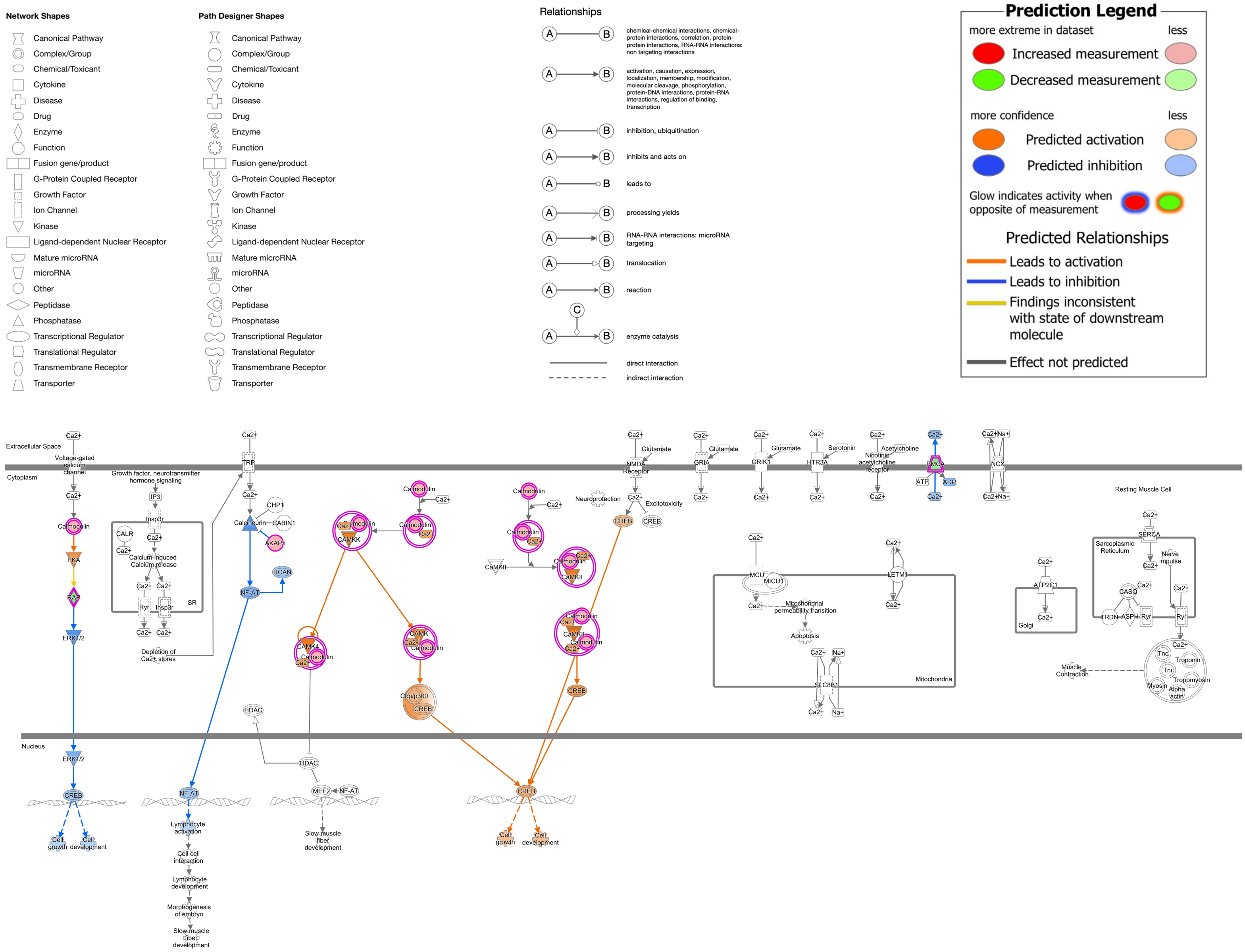

Supplementary Figure 7. Canonical Pathway of calcium signaling of the susceptible females compared to the resilient females. This figure was obtained from IPA and integrates both measured expression changes and predicted molecular activity. Nodes are color-coded to reflect the direction and magnitude of expression changes, with red and pink indicating increased expression and green and light green indicating decreased expression, where darker shades represent more extreme values. Predicted activation and inhibition are represented by orange and blue nodes, respectively, with lighter shades indicating lower confidence. Glow effects around nodes denote activity opposite to the measured direction, suggesting potential compensatory mechanisms. Edges between nodes represent predicted molecular relationships: orange lines indicate activation, blue lines indicate inhibition, yellow lines highlight inconsistencies between predictions and downstream molecular states, and gray lines represent relationships with no predicted effect.

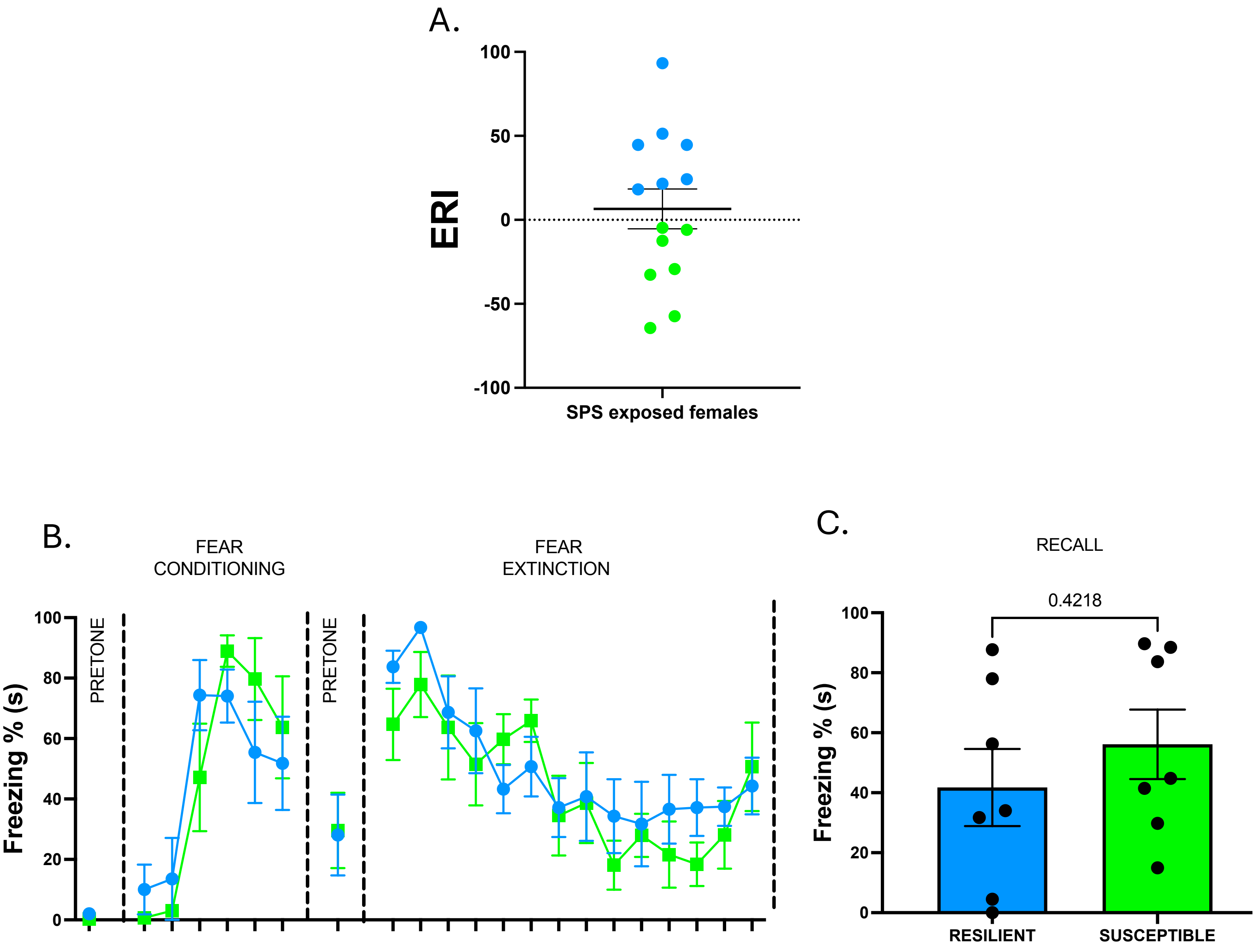

Supplementary Figure 8: Behavioral performance of resilient and susceptible females used for IL histology of NGRN and MAPT. (A) Extinction Retention Index Scores for females exposed to SPS. (B) Freezing levels of auditory fear conditioning and extinction training in females exposed to SPS, (Resilient n=7) vs (Susceptible n=7). Two-way ANOVA, Uncorrected Fisher's LSD $p<0.05$ (C) Freezing levels of extinction recall in resilient and susceptible animals. Unpaired T-test , $p<0.05$

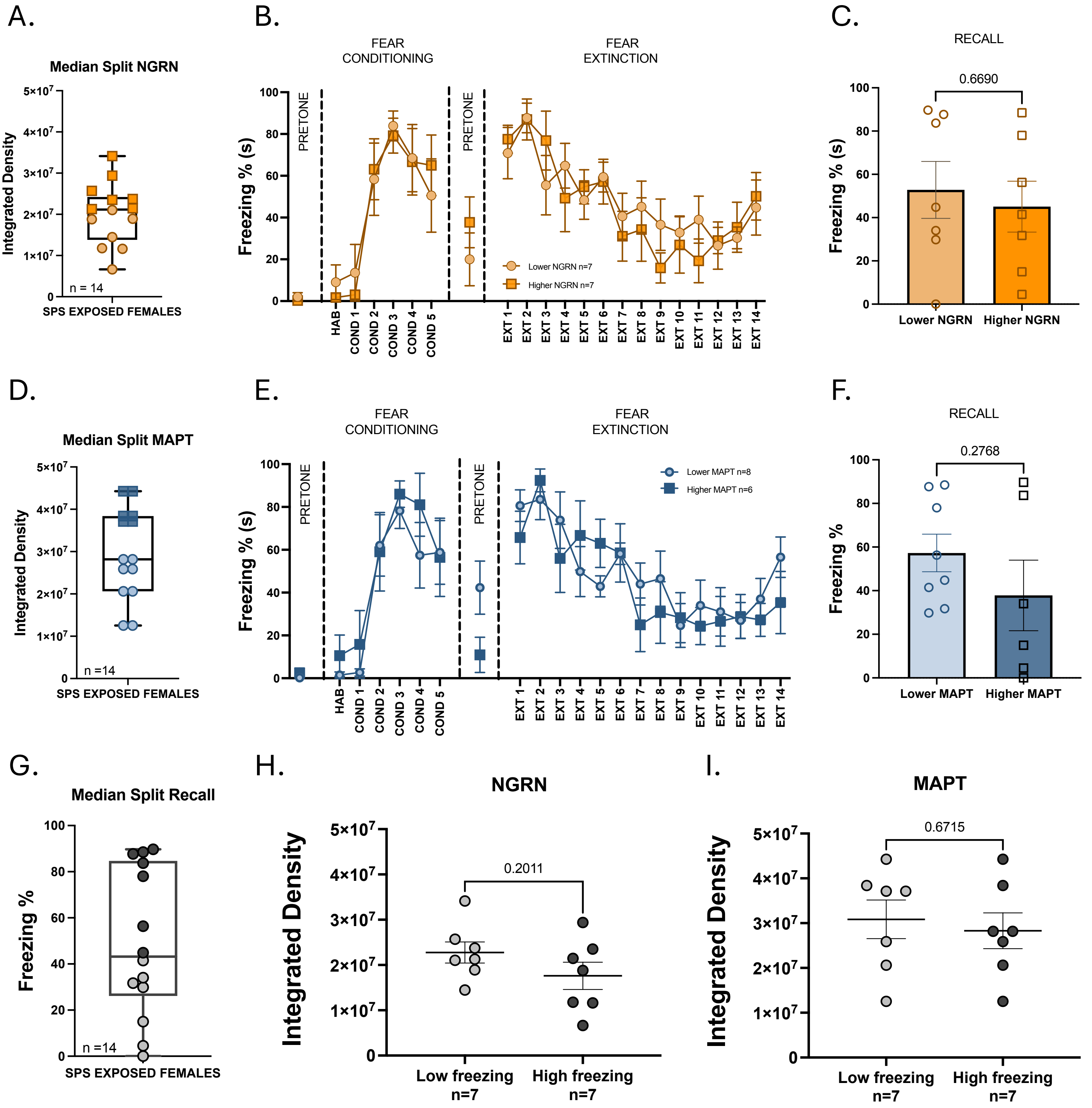

Supplementary Figure 9: NRGN and MAPT expression do not differ by extinction recall performance in the the independent immunohistochemistry cohort. (A) Animals were stratified into lower- and higher-NGRN expression groups using a median split within the cohort. (B) Freezing levels of auditory fear conditioning and extinction training in females with lower NGRN (n=7) and higher NGRN expression (n=7) Two-way ANOVA, $p<0.05$ (C) Freezing levels of extinction recall in lower NGRN (n=7) and higher NGRN expression . Unpaired T-test , $p<0.05$ (D) Animals were stratified into lower- and higher-MAPT expression groups using a median split within the cohort. (E) Freezing levels of auditory fear conditioning and extinction training in females with lower MAPT (n=8) and higher MAPT expression (n=6) Two-way ANOVA, $p<0.05$ (F) Freezing levels of extinction recall in lower NGRN (n=7) and higher NGRN expression. Unpaired T-test , $p<0.05$. (G) Animals were stratified into lower- and higher-freezing at recall expression groups using a median split within the cohort. (H) Comparison of NGRN and (I) MAPT integrated density in IL between low freezing (n=7) and high freezing (n=7). Unpaired t-test, $p<0.05$.

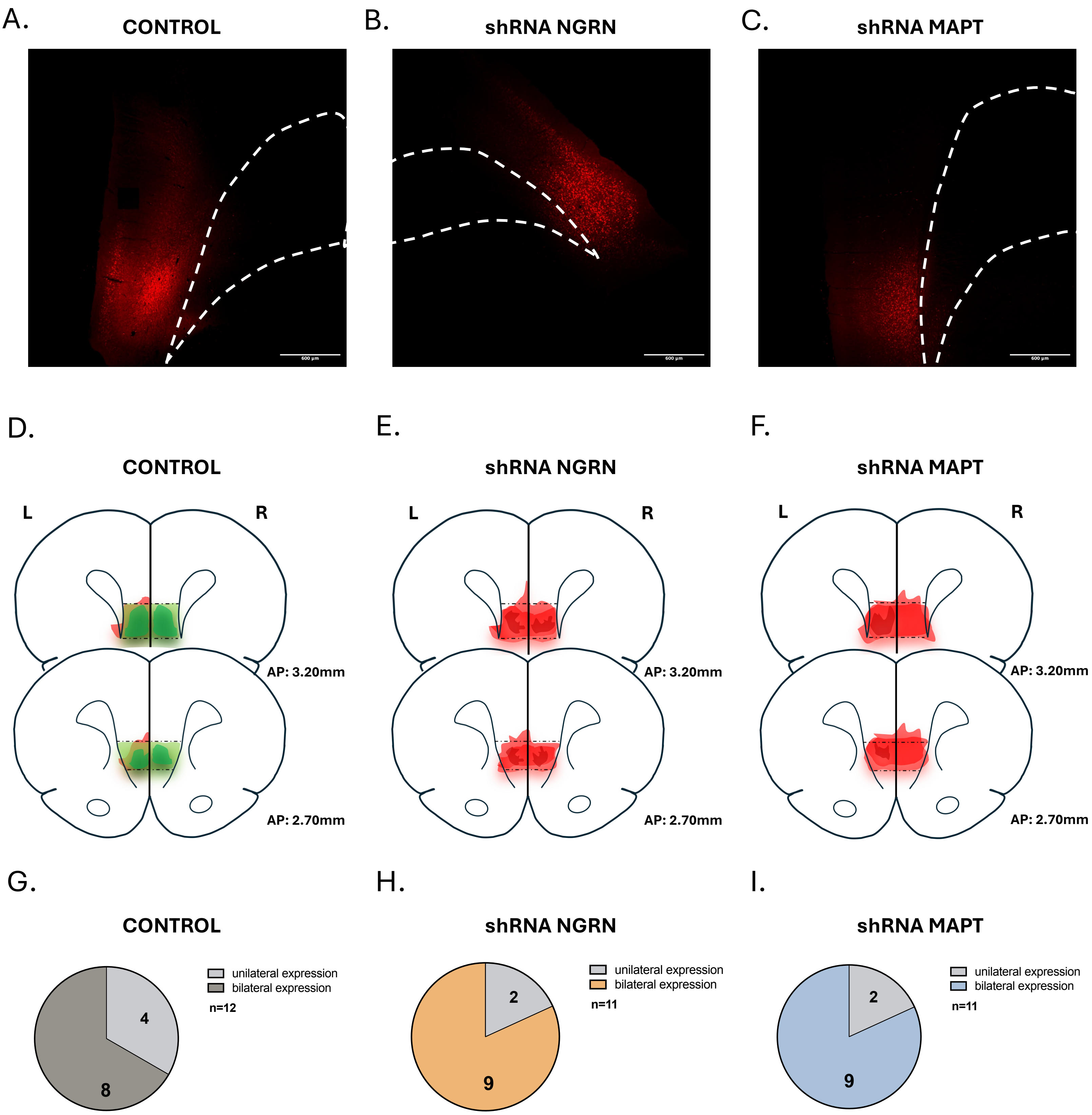

Supplementary Figure 10: Viral spread and inclusion criteria for IL-targeted Injections. Representative coronal sections showing mCherry fluorescence in the infralimbic cortex for (A) control (B) shRNA NGRN and (C) shRNA MAPT. Dashed white lines outline corpus callosum, Scale bar = 600μm. Schematic viral spread across infralimbic cortex in (D) control (E) shRNA NGRN and (F) shRNA MAPT animals. Colors denote the spread of flourescence across animals in each group. Lighter intensity represents greatest spread, medium intensity corresponds to average expression observed and dark intensity indicated unilateral expression. Pie charts showing viral expression across hemispheres in (G) control (H) shRNA NGRN and (I) shRNA MAPT groups. Numerical values inside each segments indicated number of animals.

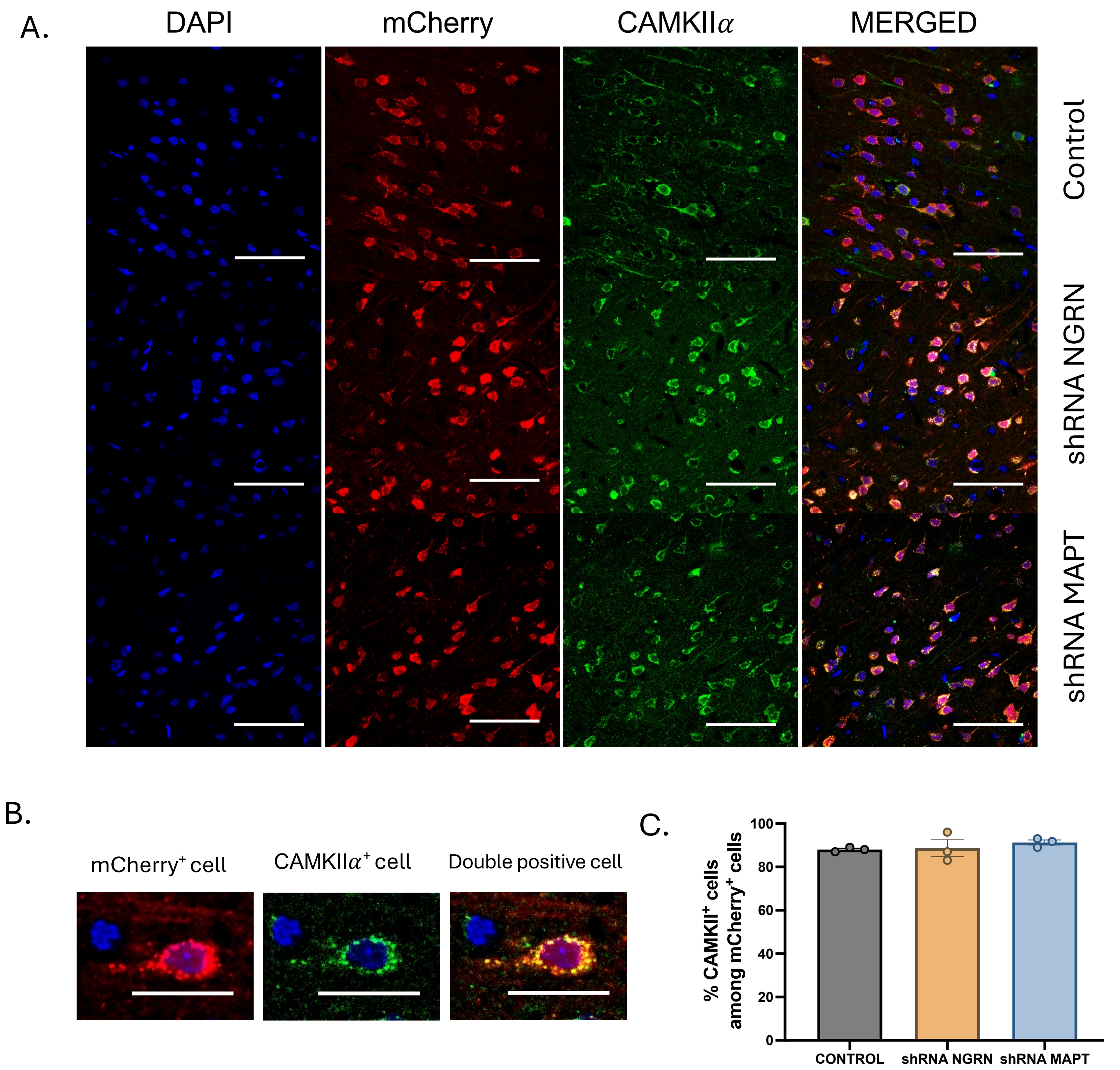

Supplementary Figure 11: Verifying viral expression in CAMKIIa-expressing neurons in IL. (A) Representative images of the infralimbic cortex showing nuclear staining (DAPI), viral expression of mCherry (red), CAMKII immunolabeling (green) and their corresponding merged image. Images show a robust viral expression and overlap with CAMKII-positive cells. Scale bar = 60 µm. (B) Inset of an individual cell showing DAPI-based inclusion criterion for an mCherry$^+$ cell and a CAMKII$^+$ cell. The merged image represents a double positive cell. Scale bar = 20 µm. (C) Percentage of infected neurons that were CAMKII$^+$ calculated as (double positive cells/ total mcherry$^+$ cells) x 100. Individual points represent animals. Control n=3 , shRNA-NGRN n=3 and shRNA-MAPT n=3
